# Sexual Dimorphism and Aging in the Human Hyppocampus: Identification, Validation, and Impact of Differentially Expressed Genes by Factorial Microarray and Network Analysis

**DOI:** 10.3389/fnagi.2016.00229

**Published:** 2016-10-05

**Authors:** Daniel V. Guebel, Néstor V. Torres

**Affiliations:** ^1^Biotechnology Counselling ServicesBuenos Aires, Argentina; ^2^Systems Biology and Mathematical Modelling Group, Departamento de Bioquímica, Microbiología, Biología Celular y Genética, Facultad de Ciencias, Universidad de La LagunaSan Cristóbal de La Laguna, España

**Keywords:** hippocampus, aging, sexual differences, microarray, senescence, mitochondria, autophagia, microRNAs

## Abstract

**Motivation:** In the brain of elderly-healthy individuals, the effects of sexual dimorphism and those due to normal aging appear overlapped. Discrimination of these two dimensions would powerfully contribute to a better understanding of the etiology of some neurodegenerative diseases, such as “sporadic” Alzheimer.

**Methods:** Following a system biology approach, top-down and bottom-up strategies were combined. First, public transcriptome data corresponding to the transition from adulthood to the aging stage in normal, human hippocampus were analyzed through an optimized microarray post-processing (Q-GDEMAR method) together with a proper experimental design (full factorial analysis). Second, the identified genes were placed in context by building compatible networks. The subsequent ontology analyses carried out on these networks clarify the main functionalities involved.

**Results:** Noticeably we could identify large sets of genes according to three groups: those that exclusively depend on the sex, those that exclusively depend on the age, and those that depend on the particular combinations of sex and age (interaction). The genes identified were validated against three independent sources (a proteomic study of aging, a senescence database, and a mitochondrial genetic database). We arrived to several new inferences about the biological functions compromised during aging in two ways: by taking into account the sex-independent effects of aging, and considering the interaction between age and sex where pertinent. In particular, we discuss the impact of our findings on the functions of mitochondria, autophagy, mitophagia, and microRNAs.

**Conclusions:** The evidence obtained herein supports the occurrence of significant neurobiological differences in the hippocampus, not only between adult and elderly individuals, but between old-healthy women and old-healthy men. Hence, to obtain realistic results in further analysis of the transition from the normal aging to incipient Alzheimer, the features derived from the sexual dimorphism in hippocampus should be explicitly considered.

## Introduction

It is increasingly recognized that many organs and tissues exhibit sexual dimorphic features (Yang et al., 2006; Coriati et al., 2014; Liu et al., 2015; Gallelli et al., 2016; Jašarević et al., 2016; Li et al., 2016; Seo et al., 2016). In this regard the brain is not an exception (Nance and Gorski, 1975; Cooke and Woolley, 2005; Berchtold et al., 2008; Joel et al., 2015; Mottron et al., 2015; Renoir et al., 2015; Marques et al., 2016). Interestingly, the primary specification of the gonads during embryogenesis is anticipated by the earlier expression of sexual dimorphism in the brain (Dewing et al., 2003).

When dealing with aging and neurodegenerative diseases, such as Alzheimer's disease, the effects of sexual dimorphism overlap those caused by the normal aging process. As aging is the main risk factor for the appearance of “sporadic” Alzheimer (more than 95% Alzheimer's cases), it is of key importance to guarantee the possibility of unambiguously identifying the mode of action of the genes involved. This can be achieved by assessing both their “combined effects” (interaction effects) as well as their “pure effects” (independent effects).

Although sexual dimorphism in human hippocampus was recognized some years ago (Berchtold et al., 2008), little attention has been paid to this fact in the further analysis of the evolution from aging to Alzheimer's Disease (Berchtold et al., 2010, 2014; Cribbs et al., 2012; Prieto et al., 2015).

On the other hand, most of the information about sexual dimorphism has been obtained through high-density oligonucleotide microarrays. However, it is well-known that this technique has several drawbacks, both at the analytic and at the post-processing levels (Guarnaccia et al., 2014; Yang et al., 2014; Chrominski and Tkacz, 2015). In order to overcome some of these limitations, we have developed Q-GDEMAR, an alternative methodology for microarray data post-processing (Guebel et al., 2016). Among other positive features, Q-GDEMAR allows a high sensitivity for the detection of genes differentially expressed while maintaining low levels of false discovery rate (FDR).

In this work we will focus on the analysis of data about sex dimorphism and aging in the human hippocampus produced by Cotman's Group (Berchtold et al., 2008). Several reasons lead us to work with these data: (i) data are deposited in a public repository; (ii) analyses were performed with a technology still in use (Affymetrix HG-133plus2.0 instead of the older Affymetrix HG-133 as in other studies), thus allowing to analyse the complete genome; (ii) although herein we restricted our analysis to the hippocampus, the original study mapped several brain regions. Hence, the present study can be further replicated to the other regions as well; (iii) there is a historical continuity in the study of Alzheimer pathology by the Cotman's Group. Hence, these data can be further compared with experimental series belonging to the stages of Mild-Cognitive Impairment (MCI) and Advanced Alzheimer which were processed by the same technology.

For this purpose we will make use of the Q-GDEMAR method, but coupled to factorial analysis. Moreover, although a “summarization operation” is usually applied as last step of the microarray data processing (Irizarry et al., 2003a,b; McCall et al., 2010), we decided to skip this step in order to optimize these analyses. In fact, there are several assumptions implicit in summarization that are actually not fulfilled, thus leading to false inferences (see Section Microarray Summarization).

Equipped with the methodology of analysis commented above, we deal with the identification and validation of the genes in human hippocampus whose expression depends on the age, the sex, and the age-sex interaction. For each one of these groups of genes identified we build compatible networks, and subject them to further ontology analyses. Finally, we assess the impact of sexual dimorphism on four important neurodegeneration-related aspects: the phenomena of cellular aging and senescence, mitochondrial functioning, autophagy-mitophagy, and microRNA expression.

## Materials and methods

### Microarray data

Hippocampal transcriptional data (Berchtold et al., 2008) were retrieved from the Gene Expression Omnibus database (accession code: GSE11882, www.ncbi.nlm.nih.gov/geo). Data come from post-mortem samples of 47 individuals. The samples were analyzed by Affymetrix HG133plus2.0 microarray. Based on the available metadata, the individuals were stratified by considering 64 years-old as cut-off value. In addition, the samples were also classified according to the gender, thus resulting in four final conditions: “Younger Men Group” (*n* = 9, age median = 28 years; age range = 20–45 years), “Younger Women Group” (*n* = 9, age median = 44 years, age range = 26-64 years), “Older Men Group” (*n* = 15, age median = 83 years, age range = 69–97years), and “Older Women Group” (*n* = 14, age median = 82.5 years, age range = 70–99 years).

### Microarray post-processing and full factorial

The log_2_-transformed data were submitted to a post-processing elaboration by the Q-GDEMAR method (Guebel et al., 2016). The method performs a computational deconvolution of the central region of the data distribution. The parametric characterization of this region in terms of a Gaussian distribution provides narrower limits to the genes whose expression fluctuates only stochastically. The comparison of these limits with the overall data distribution finally allows to determine with greater sensitivity and lower FDR, what probes are being differentially hybridized.

In the present work Q-GDEMAR has been extended to include a factorial design (Montgomery, 2004). In fact, the adopted classification of the samples follows an experimental design comprising two variables (A, Age; B, Gender), where each variable has two levels: −1 (low level) and +1 (high level). If A^+^ = Older and A^−^ = Younger, while B^+^ = Women and B^−^ = Men, the correspondence between the experimental design and the four groups previously defined can be seen as follows: A^+^B^+^ = Older Women Group, A^+^B^−^ = Older Men Group, A^−^B^+^ = Younger Women Group, and A^−^B^−^ = Younger Men Group.

In a 2 × 2 factorial design, the “expected” level (Ŷ) of a given microarray probe *k*, for an individual with *i* years of age, and *j* codified gender, can be accounted for by a bi-linear model such as that shown in Equation (1), where μ, α, β, and γ are model parameters computed as regression coefficients. In particular, the γ coefficient is the “interaction coefficient.”

$$\begin{array}{c} {Ŷ}_{i,j,k}=\mu +\alpha \;[\text{Age}]\;+\beta \;[\text{Gender}]+\gamma \;[\text{Age}]\;[\text{Gender}] \end{array}$$

Alternatively, we will focus on the “interaction effect” due to its close relation with the coefficient γ as is shown in Equation (2), where *M* is the median value of the groups indicated.

$$\begin{array}{l} Interaction\;Effect=\left(\frac{1}{2}\right)\gamma =0.5\;[\left(M_{\left(A^{+}B^{+}\right)}+{\text{M}}_{\left(A^{-}B^{-}\right)}\right)\\ \;-\left(M_{\left(A^{+}B^{-}\right)}+M_{\left(A^{-}B^{+}\right)}\right)] \end{array}$$

When the “interaction effect” is significant, it means that the analyzed variables do not operate independently, and their effect will depend on the combination of the specific values taken by the variables in each condition. In the present case, where sexual dimorphism is analyzed, a significant interaction effect means that some sets of genes may be expressed differentially in males or females, but the name-list of these differential genes will be conditioned by the range of age to which each individual belongs (Younger or Older Groups).

In the microarray analyzed herein we worked with 43747 annotated probes. This produced a list containing some thousands of interacting genes, making the individual interpretations difficult, because the interaction effects depend on the combination of four values (see Equation 2). Thus, we computed a new coefficient (Super-Ratio; see Equation 3) together with the interaction effect to facilitate the interpretation.

$$Super\;Ratio\;=\;\frac{\left(\frac{M_{Old\left(females\right)}}{M_{Young\left(females\right)}}\right)}{\left(\frac{M_{Old\left(males\right)}}{M_{Young\left(males\right)}}\right)}\;=\;\frac{Age\;Ratio_{\left(females\right)}}{Age\;Ratio_{\left(males\right)}}$$

In Equation (3), *M* also denotes the median value of the expression for the indicated groups. The “Super Ratio” correlates well (*R*^2^ = 0.7618, *n* = 2000) with the “interaction effect” (see Figure S1). This means that the super-ratio coefficient captures most of the information contained in the interaction coefficient, being in fact a measurement of the Age Effect corrected for sex. A super-ratio value around 1 is equivalent to an interaction effect around zero. However, because correlation of super–ratios was good but not perfect, we used the values of the interaction effects to determine the set of significant interactions. But we have used the super-ratio values to interpret the individual genes involved.

Moreover, in order to maximize the contrast between augmented and decreased super-ratios, those values of super-ratio comprised between 0 and 1 were transformed as Super-Ratio^*^ = −(1/Super-Ratio). Thus, the scale of super-ratios finally covered two discontinuous intervals: from −∞ to the left significant super-ratio threshold (for the diminished super-ratios) and, from +∞ to the right significant super-ratio threshold (for the increased super-ratios). In this way, interactions and super-ratios kept the same sign.

The factorial analysis, besides computing the interaction effects, also allows to determine the independent effects of the variables. Hence, the independent effect of aging is computed herein by the so-called Age Ratio^#^, which is defined as the quotient between the median[log_2_(signal)_Older Group_] and the median[log_2_(signal)_Younger Group_]. Likewise, the independent effect of sex is computed by the so-called Sex Ratio^#^, which is defined as the quotient between the median[log_2_(signal)_Women_Group_] and the median[log_2_(signal)_Men_Group_].

Note that, Age Ratio^#^ and Age Ratio (Equation 3) are operationally different. The former is a unique value for each gene, covering the relation of sampled signals between those coming from the collection of all the older-individuals pooled and those coming from the collection of all the younger-individuals pooled. That is, Age Ratio^#^ is independent of sex, whereas the Super Ratio needs of two sex-dependent age ratios [Age Ratio_(females)_ and Age Ratio_(males)_]. Moreover, because age and sex have equal influence on the interaction, the Super-Ratio (Equation 3) can also be equivalently computed as a quotient of two sex ratios corrected by age [i.e., Super Ratio = Sex Ratio_(Older Group)_/Sex Ratio_(Younger Group)_]. Even in this case, sex ratios in the Super-Ratio have a scope different from that of Sex Ratio^#^ because this latter has a unique value for each gene and is age-independent.

### Microarray summarization

The current practice of microarray analysis is that the “normalization” of the data (Eijssen et al., 2013; García, 2015), is followed by a final step of data “summarization” (Irizarry et al., 2003a,b; McCall et al., 2010). This process is based on the Tukey's median polishing algorithm (Sposito, 1989). Accordingly, a value to represent each probe-set is computed as weighted average of the inverse of distance of the individual probes with respect to the median value of the series. Thus, summarization is a resource to convert the measurements of the individual probes within a probe-set into a numeric assignation, from which the gene status is inferred.

At first glance, the summarization operation seems reasonable. However, we did not summarize our microarray data because this operation has some important shortcomings, many of them derived from the fact that its underlying assumptions are not fulfilled.

Some described summarization troubles are the following: (i) The method is prone to generate numerical artifacts (Giorgi et al., 2010); (ii) The hybridizing-probes used for the measurements only recognize short segments of the target sequence. As a result, some of the probes within a set-probe can exhibit cross-reactivity toward other targets (Chou et al., 2004); (iii) Probes within a same probe-set don't behave with the same efficiency, either in hybridization or in binding to the tracer molecule. Some probes within a probe-set show changes in opposite directions (Jayaraman et al., 2007; Suzuki et al., 2007); (iv) Genes equipped with different promoters can generate different isoforms, and these transcripts are affected differentially by the post-transcriptional processes (D'Mello et al., 2006; Miller et al., 2008); (v) Even in the case of a unique transcript, more than 60% of human genes have a variable number of exons. This generates different “spliced” transcripts that usually have different degrees of hybridization to a given probe (Moffa et al., 2004; Salisbury et al., 2009; Tollervey et al., 2011; Baj et al., 2013). Finally, in dealing with summarization it has been claimed as necessary to refine the detection of “outliers” within the probe-set by Dixon's Test (Jaksik et al., 2009). But this is not possible from the statistical point of view, because this test requires larger number of replicates to be valid. More importantly, excluding some probes from a probe-set due to their “atypical” behavior while averaging the remaining members, can mask but not solve the intrinsic physical, chemical, and/or biological peculiarities evidenced by some microarray probes.

### Network and ontology analyses

**Figure 1:**
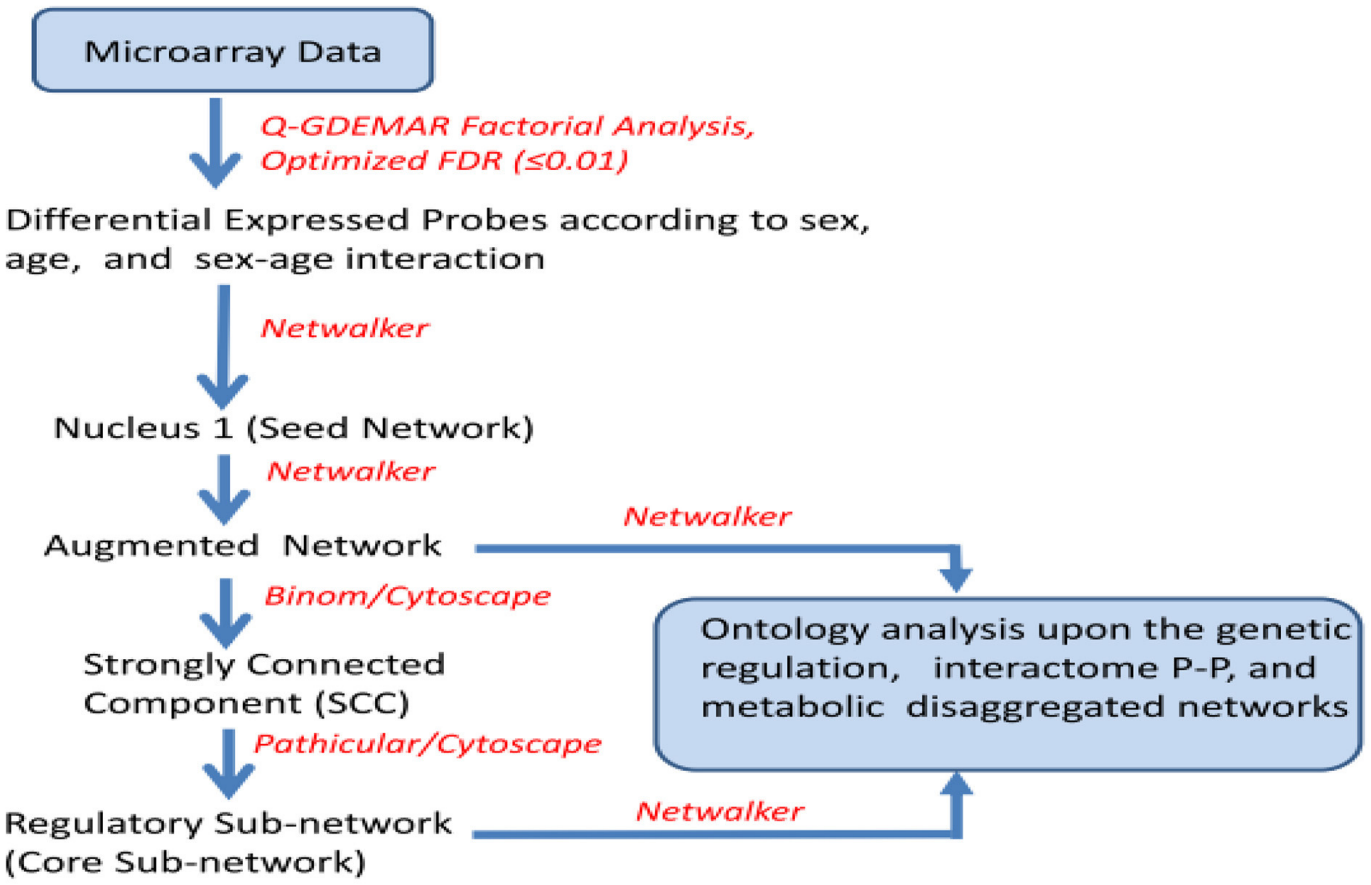
**Sequence of operations performed upon the microarray data to achieve separated networks corresponding to genetic regulation, protein-protein interactions (PP), and metabolic regulation at two different levels of complexity (augmented and core network), for each one of the conditions analyzed (sex ratio positive and negative, age ratio positive and negative, and interaction positive and negative)**.

The set of probes identified as differentially expressed for each one of the factors analyzed (interaction, age, and gender) were classified as up- and down-regulated, and then subjected to network analysis (Netwalker 1.0; Komurov et al., 2010, 2012). The directly inferred network obtained from the differentially expressed genes (nucleus) was used as a base to build upon an “augmented” network. This was constructed with molecular nodes which, though undetected, were inferred to exist, supported by evidences of interaction with the genes measured experimentally (see Figure 1).

The number of nodes added with this strategy is a minor fraction of the total. However, some of the isolated modules can be interconnected through augmentation, thus contributing to enrich the ontology analysis. In some cases the added nodes act as “hub” nodes, and hence they become more relevant than the experimentally detected nodes.

Given that the associated networks inferred from Q-GDEMAR were large, the interactions within the networks were filtered to isolate the sub-network so-called “strongly-connected component” (SCC; Dorogovtsev et al., 2001). This was done by using Binom (Bonnet et al., 2013). In turn, the SCCs were further reduced to a minimal core of dense, regulatory sub-networks by using Pathicular (Joshi et al., 2010). Finally, both the augmented and reduced-size networks were subjected to ontology analysis (Netwalker). In some cases of interest, the structural and ontology analyses were done by discerning between genic, protein-protein, and metabolic interactions separately for each one of analyzed factors.

## Results and discussion

### Factorial analysis

**Figure 2:**
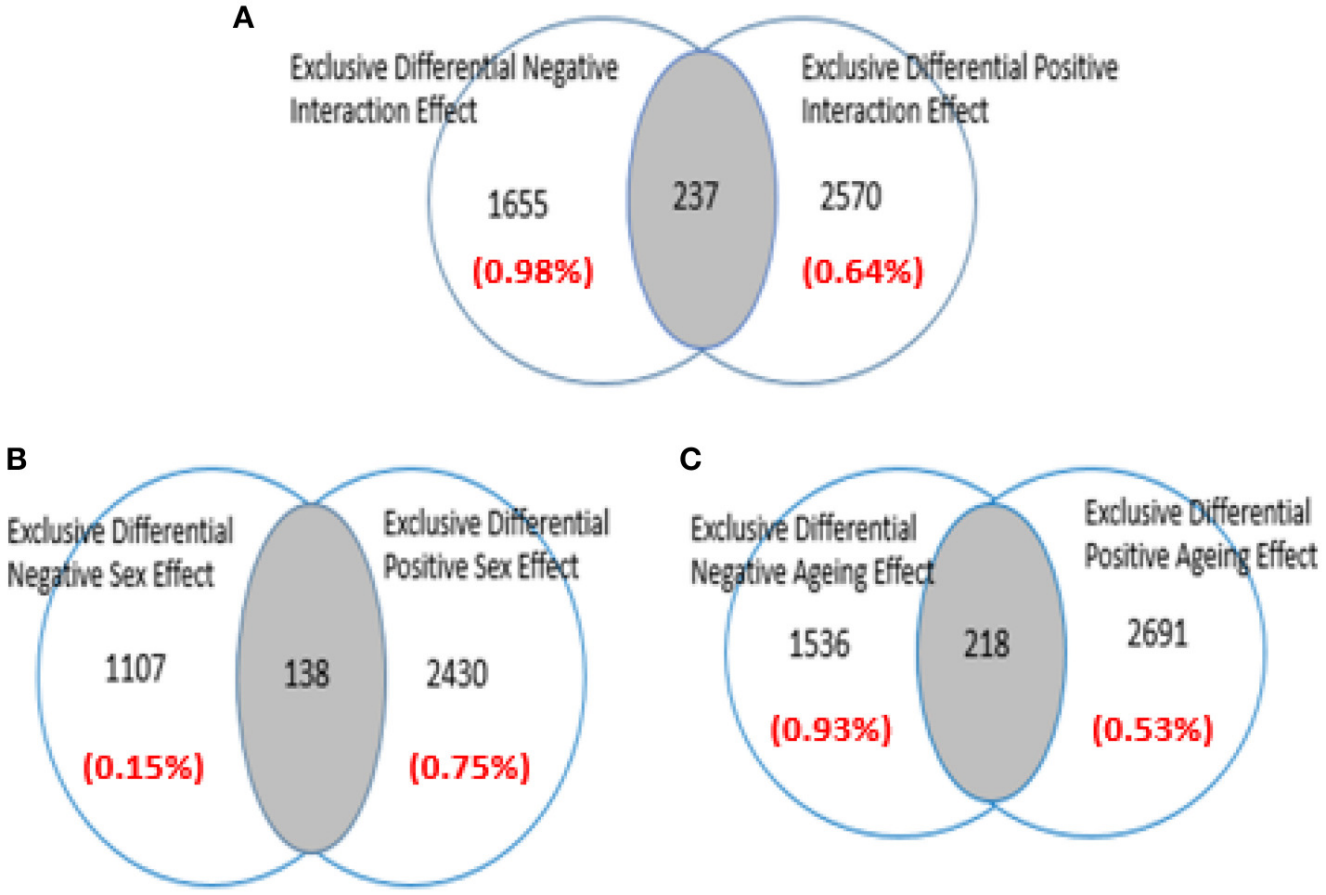
**Venn diagram showing how the data of microarray GSE11882 (Berchtold et al., 2008) are disaggregated by Q-GDEMAR (Guebel et al., 2016)**. The factorial micro-array analysis allows to identify; **(A)** Genes operating under the age-sex interaction mode; **(B)** Genes operating under the sex-dependent mode; **(C)** Genes operating under the age-dependent mode. The values between parentheses indicate the level of False Discovery Rate (FDR) associated to each class of genes.

Figure 2 show the three sets of genes with differential expression that were identified in the analyzed microarray: the associated with aging only; the associated with gender only, and those simultaneously related with gender and age (i.e., the interaction-dependent genes). For complete details see Table S1 (Supplementary Materials).

From these results, the following relevant features arise: (a) Low values of FDR were achieved across all the tested conditions; (b) The number of genes up-regulated in the Women Group was in 2:1 ratio with respect to the ones with increased expression in the Men Group (see Figure 2B); (c) In the Older Group the number of genes up-regulated was 75% greater than the number of genes down-regulated (see Figure 2C); (d) A minor number of intersections occurred between the opposing categories tested (shaded areas in Figures 2A–C).

The importance of the low FDR values is that they have been achieved without compromising the length of the lists of differential genes. Moreover, the FDRs computed are not referred to individual genes but covering the entire block of each class of genes. The second point, could reflect the higher complexity of the biological responses present in the Women Group with respect to the ones in the Men Group. The importance of the third point is given by the fact that aging is viewed mainly as the loss of several adulthood functionalities. Our results, however, suggest the occurrence of a genetic “program of aging” with its own features. They could also reflect the presence of a very intense “compensatory response” (Bartsch and Wulff, 2015). In any case, these results go in a direction opposite to the previously reported for the hippocampus (Berchtold et al., 2008). These researchers, due to an extremely conservative pre-processing treatment, have lost 64% of their original data and consequently, the diminution of false positives was forced at the cost of the increment of false negatives.

Concerning the intersections observed with the age- and sex-dependent genes, these are generated due to the presence of different probes corresponding to the same gene in the contrary groups (Male/Female or Older/Younger). Similarly, the intersection at the interaction-dependent genes reflects a situation in which a same gene has more than one probe in each factor, and interactions occur between these multiple combinations of probes. We ruled-out that intersections at the interactions could reflect the occurrence of false positives. In fact, the incidence ratio derived from these cases is one order of magnitude higher than the (weighted) average FDR computed for the interaction genes (5.61 vs. 0.77%).

We concluded that factorial Q-GDEMAR can disaggregate the genes in relation to their factor-dependences in a consistent manner. In addition, is verified that avoidance of the microarray summarization does not compromise the extent of the differential genes detected. Actually, not summarizing might minimize the loss of false negatives occurring in the current methods, thus diminishing the possibility of discarding genes that could be relevant in the further steps of the analysis performed herein.

#### Age and sex interaction effects

**Figure 3:**
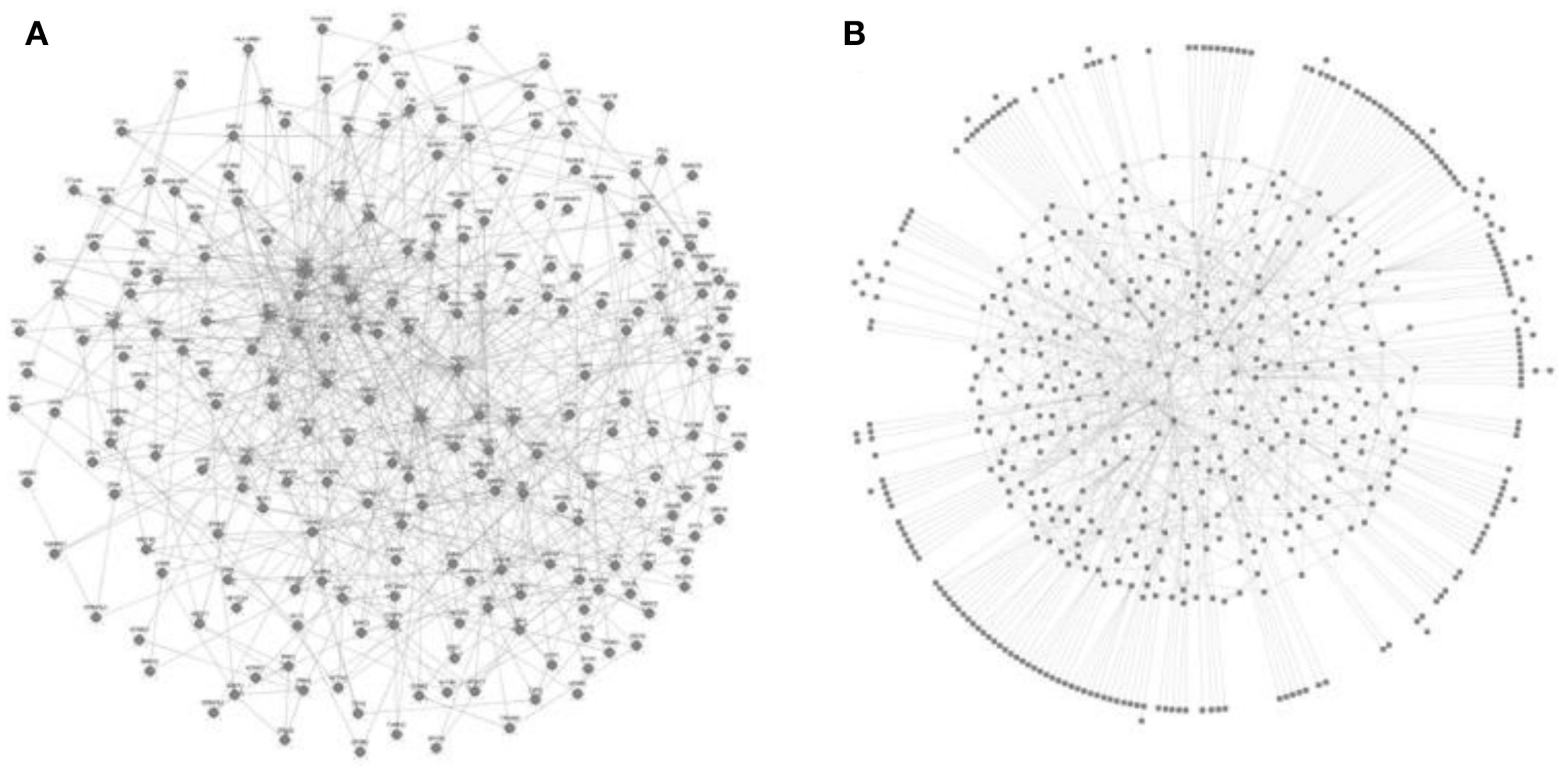
**Networks isolated as strongly connected components (SCC) from the Protein-Protein (P-P) connections identified in nucleus 1 under the interaction mode**. The nucleus 1 is the network that arises from known inter-relationships between nodes without need to add any additional connector to the list of differential genes detected. **(A)** Case of positive interaction (*n* = 229 nodes); **(B)** Case of negative interaction (*n* = 549 nodes).

Figure 3 shows the protein-protein (P-P) interactions network obtained from the analysis of the probes for the case of interaction-dependent genes. This type of networks include all type of physical interactions and chemical transformations implied in signal transduction and/or the formation of homo- or hetero-multiprotein complexes (De Las Rivas and Fotanillo, 2010). Since the microarrays actually allow to determine the relative level of the transcripts but not the level of their corresponding proteins, these networks are “potential” in the sense that they are just compatible with the list of transcripts detected.

It is worth to comment that the networks underpinning the positive and negative interactions are actually minor functional units of their corresponding complete networks. Importantly, they have quite different composition even when details of their topology cannot be discerned visually in Figure 3 due to their compact density. Accessing to the composition of these sub-networks allowed us to determine their ontology, which in Table 1 clearly shows the occurrence of significant functional differences between genes operating under positive or negative interaction.

**Table 1:** **Main functionalities derived from the ontology analysis practiced on the strongly connected components (SCC) corresponding to the interaction-dependent mode (for details see the Table S2)**.

**P-P under Negative interaction**		**P-P under Positive interaction**	
**Ontology process**	Hypergeometric probability^*^	**Ontology process**	Hypergeometric probability^*^
NGF signaling	5.1 × 10^−8^	NGF signaling	3.3 × 10^−14^
Positive regulation of anti-apoptotic-mechanisms	1.4 × 10^−5^	Positive regulation of anti-apoptotic-mechanisms	4.1 × 10^−8^
Induction of apoptosis (Neuron, B-cells)	2.1 × 10^−4^	Induction of apoptosis (Endothelial cells)	5.9 × 10^−6^
Coagulation, haemostasis	1.4 × 10^−7^	Regulation cell cycle arrest (G1/S, G2/M, Mitosis)	3.4 × 10^−6^
Synaptosome	1.4 × 10^−6^	Dendrite	2.9 × 10^−8^
Positive regulation of GABAergic transmission	1.2 × 10^−4^	Axogenesis, axon guidance	8.1 × 10^−7^
Cell senescence	2.5 × 10^−4^	Negative regulation cell communication	2.1 × 10^−6^
Cell-cell adherens junctions	1.5 × 10^−5^	Memory	2.5 × 10^−6^
Response to oxygen level	1.3 × 10^−3^	Response to hypoxia	8 × 10^−6^
Endothelial proliferation, and migration. Blood Brain Barrier establishment	2.5 × 10^−2^	Endothelial cell differentiation and migration	5.1 × 10^−3^
Response to UV and radiation. DNA damage	1.9 × 10^−3^	p53 binding	1.9 × 10^−6^
Negative regulation of IGFR signaling	3 × 10^−6^	Wnt receptor signaling	3.4 × 10^−6^
Cytoskeleton organization	1.2 × 10^−3^	Actin cytoskeleton, Spectrin	1.1 × 10^−5^
Response to steroids, androgens, progesterone	8.4 × 10^−4^	Response to insulin and nutrient level	1.9 × 10^−5^
Ribonucleotides tri- and bi-phosphate catabolism	1.1 × 10^−3^	Chaperone-mediated protein processes	2.7 × 10^−5^
Neurogenesis/Neuroblast proliferation	1.5 × 10^−2^	Plasma membrane organization, Rafts	3.1 × 10^−5^
Negative regulation of astrocytes differentiation	2.2 × 10^−3^	Sex Differentiation, Steroid response	4.2 × 10^−5^
Astrocytes migration	1.8 × 10^−4^	Nitric Oxide production, NOS1, NOS3, Arginine	4.5 × 10^−5^
Microglia activation	1.3 × 10^−3^	Inflammatory response (IL-12, IFNG)	5.9 × 10^−5^
Inflammatory response (IL6)	6.6 × 10^−4^	TGFB receptor activity	5.8 × 10^−5^
Anti-inflammatory response (IL-10, IL8)	1.1 × 10^−3^	Mononuclear cell proliferation	3.8 × 10^−7^
Inmunological response	1.1 × 10^−5^	Inmunological response	3.4 × 10^−5^

* *The probabilities values are corrected for the multi-comparisons. In addition, the values indicated are the averages of the probabilities along the several ontology categories that make up each sub-group defined, but weighted according to the relative frequency in which the initial ontology classes appeared from the analysis*.

A rapid glance at data in Table 1 could lead to the conclusion that the interaction groups compared share some ontology classes (e.g., NGF signaling, apoptotic and anti-apoptotic mechanisms, response to oxygen, and cytoskeleton), whereas other classes are differentially enriched in one or other group. However, a more detailed analysis showed that even those of apparently common ontology categories can also be considered as different.

The differences among the shared ontologies are visible at two levels. First, the molecules involved in each group are quite different even when they appear collected under a same ontology term (compare the set of molecules participating in NGF signaling, cytoskeleton, or response to oxygen for both interaction groups in Table S2). Second, there are highly significant differences between the hypergeometric probabilities associated to the same nominal ontology category. These differences can be interpreted as a variation in the “frequency” of the molecular process specified. In fact, the higher the frequency of a given type of molecular event in the network, the higher is its ontology significance, and lower the expected value of the hypergeometric probability computed for its stochastic occurrence (Komurov et al., 2010).

The probabilities shown in Table 1 are weighted-averages values, where the weighing factor used is the relative frequency of an event. The application of this concept leads us to conclude that the anti-apoptotic mechanisms operating under the negative interaction is only a one-tenth of the homolog response under the positive interaction. Therefore, as apoptosis is less frequent under the positive interaction condition it can be expected that mutations and severe disorders occurring in neurodegeneration tend to accumulate more in older women than in older men.

It is known that Nerve Growth Factor (NGF) is a potent neurotrophic factor (Fuenzalida et al., 2007). Also based on the values of the hypergeometric probabilities in Table 1, NGF receptor signaling under the negative interaction can be expected to be around 10^6^ times less than under the positive interaction condition. Supporting this inference, a senescence ontology term appeared significantly enriched under the negative interaction condition (see Table 1).

Compatible with the neuronal apoptosis referred to Table 1, the processes of neurogenesis and microgliosis appear significantly increased under the negative interaction condition. Astrogliosis occurs in the same group but probably driven by immature precursor cells, because even though astrocyte precursor migration capacity is conserved, their terminal differentiation is inhibited (see Table 1).

Note that astrocytes and neurons derive from a common precursor (Koblar et al., 1998). However, while neuroblast proliferation requires of its activation by glial Pannexin (Spéder and Brand, 2014), the differentiation of astrocytes requires of Leukemia Inhibitory Factor (LIF) and Retinoic Acid (Asano et al., 2009). In agreement with this, LIF and its receptor LIFR appear increased in the Positive Interaction Group (interaction effects of +1.99 and +2.82, respectively), as are several transcripts of genes that are induced by Retinoic Acid (RARG = +2.1, RARRES1 = +1.83, STRA13 = +1.75, STRA6 = +1.48). Further, the available data allow to corroborate that these transcripts are down-regulated in the Negative Interaction Group. Instead, the processes of neurogenesis and astrogliosis are not detected in the Positive Interaction Group, but a significant immunological response is observed, dominated by the activation of T-cells and proliferation of infiltrating monocytes (see Table 1).

In addition, some differential features are also detected in the vascular compartment. In the Negative Interaction Group, ANGPT1 (interaction = −1.28) and ANGPTL1 (sex effect = −1.83) are increased, whereas in the Positive Interaction Group, an increase in ANGPTL6 (sex effect = +1.22) is observed. Importantly, ANGPT1 acts as survival factor for endothelial cells (Milner et al., 2009), while ANGPTL1 (ANGPT3)—induced by ischemia—, exerts an anti-edema effect (Lai et al., 2008). The ANGPTL6 (ARP5) acts on stem cells favoring their self-renewal (Cui et al., 2010).

Interestingly ANGPTL4 is increased in both groups (age effect = +1.42), but only in the Positive Interaction Group an increment of Claudin 5 (CLND5, interaction = +1.6) is observed as expected (Bouleti et al., 2013). Of note, when endothelial cells are under hypoxic conditions, Claudin 5 is induced in an autocrine way by ANGPTL4. But ANGPTL4 is also abundantly produced by infiltrating monocytes (Frenzel et al., 2015), which can explain why CLDN5 is up-regulated only in the Positive Interaction Group (see Table 1).

Sprouting in the capillaries can be an activity associated to The Older-Women Group, because in addition to ANGPTL4, several markers such as NID2 (interaction = +1.47), WNT4 (interaction = +1.67), PECAM1 (interaction = +2.73), ANGPTL2 (sex effect = +1.49), ANGPT2 (age effect = +1.22) are also increased. An activity of endothelial tube formation could be also present but to a minor extent, because only a part of the required molecules is dysregulated (TNFS10B interaction = +2.44; TNF10C, interaction = +1.49).

Instead, the sprouting activity in the Negative Interaction Group is associated to the main regulators such as ANGPT1 (interaction = −1.28) and WNT7A (interaction = −3.51), together with ANGPT2 (age effect = +1.22), PECAM1 (sex effect = −1.279). In the same group, the activity of tube formation is mainly represented by the increment of ANGPT1 (interaction = −1.28) as well as by the increased transcription of IGFBP7 (interaction = −2.12), TNFRSF10A (interaction = −1.53), TNFRSF10C (sex effect = −1.54). Of note, WNT7A has been described as a critical factor for establishing the brain-blood barrier (BBB; Posokhova et al., 2015).

The differential features also extend to the basal lamina surrounding the capillaries. In the Older-Women Group the endothelium might be exposed to a probable imbalance for Laminin 5. This heterotrimeric molecule—made up by LAMA1, LAMB3, and LAMC2 subunits—shows normal level of LAMA1, but the transcription of LAMB3 and LAMC2 is increased (interaction = +1.46). Accordingly as expected from the introduced concept of interaction super-ratio, these molecules appear down-regulated in the Older-Men Group, thus compromising the functional integrity of the basal lamina.

Concerning the inflammatory activity in the endothelial cells, the NOS3 transcript—that codifies an enzyme responsible for nitric oxide (NO) production—, is up-regulated in the Positive Interaction Group (interaction = 2.12, see Table S1). It is known that NO can damage both the tight- and the gap-junctions. As a consequence, blood-brain barrier (BBB) integrity could be weaker in this group, despite up-regulated main endothelial tight junction elements (CLDN5 interaction effect = +1.60; CLDN1 sex effect = +1.28). In fact, this picture is fully consistent with the monocyte infiltration observed (Table 1). Instead, in the Negative Interaction Group there is an increased expression of several vascular anti-permeability factors, such as Angiopoietin (ANGPT), Angiomotin (AMOT), and Rho GTPase Activating Protein 35 (ARHGAP35; see Table S1).

The transcription of important molecules such as GJA1 (Connexin 43, interaction = −2.72) and TJP3 (Zona Occludens 3, interaction = −1.51) is also increased in the Negative Interaction Group (see Table S1). Given that GJA1 is mainly located in the astrocytes, a more consolidated astrocytic syncytium around the capillaries can be inferred–in individuals of the Negative Interaction Group. They might also have a higher buffering capacity for several causes of neurotoxicity (e.g., excess of glutamate or K^+^). However, it is clear that astrocytic neuroprotection is not enough to avoid the occurrence of neuronal apoptosis following NGF down-regulation in this group (see Table 1). Besides, the basal lamina—which normally localizes as a thin macromolecular layer between astrocytes and endothelium—is diminished in the Negative Interaction Group.

Moreover, the Aquaporin 4 transcript (AQP4, codes for a critical protein molecule that localizes at the astrocytic end-foot, allowing the water to drain) shows no significant changes, but is accompanied by an increased transcription of anti-sense AQP4 mRNA (AQP4-AS, interaction = −1.69). This last result, together with the occurrence of anti-permeability factors ANGPT1, AMOT, and ARHGAP35 already commented, support that hippocampus edema is not present in the Negative Interaction Group but can be present in the Positive Interaction Group.

As nitric oxide is a diffusible gas, its production in the endothelium by the isoenzyme NOS3 (interaction = 2.12) and in the neurons by the isoenzyme NOS1 (interaction = 1.76) can also damage the tight-junctions at the neighboring oligodendrocytes. There, the specific tight-junction molecule Claudin 11 (CLDN11) might not be functional due to nitrosylation, despite its up-regulation in the Positive Interaction Group (interaction effect = +1.35). The myelin wrapping the axons can become damaged as a consequence (Mayes et al., 2013).

An increased transcription of genes related with axogenesis is observed consistently only in the Positive Interaction Group (see Table 1). But, paradoxically, axogenesis was not detected in the Negative Interaction Group despite the fact that Claudin 11 is very diminished or lacking. This contradiction can be explained because Sonic Hedgehog (SHH)—that is required for both oligodendrogenesis (Boyd et al., 2015) and neurogenesis (Giacomini et al., 2015)—, actually is up-regulated in the Negative Interaction Group (interaction = −1.83). Hence, the diminution of SHH in the Positive Interaction Group might be the main cause for the need of axogenesis, while the functional deficit of Claudin 11 would only contribute secondarily. What characterizes the Negative Interaction Group from the point of view of signal transmission, is not axogenesis, but the increased expression of genes related to the synaptosome, the GABAergic process being particularly favored (see Table 1).

Finally, the interaction-dependent genes also differ in the overall inflammatory aspect. NO is mainly produced under the Positive Interaction condition. In this group, the inflammatory signature is driven by the production of IL-12 (see Table 1). It is known that IL-12 increases the Th1 subpopulation of T lymphocytes and natural killer (NK) cells. While once activated, the former also leads to the production of the inflammatory cytokine IFNG (see Table 1), the latter might lead to a direct cytotoxic effect. The fact that IL-12 is produced by antigen-stimulated cells (i.e., macrophages) is also corroborated consistently by results shown in Table 1.

Instead, the constellation of cytokines in the Negative Interaction Group comprises both inflammatory (IL-6) and anti-inflammatory molecules (IL-10, IL-8). This could reflect that inflammation is being balanced. In fact, IL-10 exerts an effect opposite to that of IL-12. By diminishing the Th1-cell populations, the production of inflammatory cytokines (TNF-alpha, IL-1beta, and IL-12) also falls, as well as the molecules involved in antigen presentation (MHC-class II). Consistently, although no variation in TNF-alpha is detected, many closely related molecules (TNAP8L2, C1TNF4, TRAF6, TRAF7, BRE, TNFRSF10C, and TNFAIP8L2) are up-regulated only in the Older-Women Group (interaction effect ranged between +1.60 and +2).

#### Effects of aging (sex-independent)

It was already shown that 75% more differentially up-regulated genes were detected in the Older Group compared to the Younger Group (Section Factorial Analysis). Important dissimilarities in the hippocampal physiology underlie these differences, as can be verified from the ontology analyses performed (see Table 2 and Tables S3–S6). According to the definition of Age Ratio^#^ (Section Microarray Post-processing and Full Factorial), when the values of Age Ratio^#^ in Table 2 (or in Table S4) are significantly higher than the unity, this means that the transcription of the genes involved is up-regulated in the Older Group (or diminished in the Younger Group). On the other hand, when the values of Age Ratio^#^ are significantly less than the unity, this means that the transcription of the genes involved is up-regulated in the Younger Group (or down-regulated in Older Group).

**Table 2:** **Main functional classes at the protein-protein (P-P) level prevailing by the Age Ratio^#^ discriminating criterion**.

**Ontology process**	**Genes with age ratio^#^ < 1**	**Genes with age ratio^#^ >1**
	Hypergeometric Probability^*^	Hypergeometric Probability^*^
Anti-apoptotic mechanisms	4.4 × 10^−4^	5.2 × 10^−8^
Apoptosis induction	4.1 × 10^−2^	2.6 × 10^−4^
Platelet activation, blood coagulation	7.8 × 10^−3^	9.7 × 10^−7^
NGF signaling	1.3 × 10^−3^	1.6 × 10^−7^
Laminin 5 and 2 Complex	3.0 × 10^−6^	
Response to hypoxia	1.9 × 10^−2^	8.5 × 10^−6^
Kainate selective glutamate receptors	3.4 × 10^−2^	1.1 × 10^−3^
Axogenesis, axon guidance	NDAS^**^	1.9 × 10^−3^
Dendritic spines	NDAS	2.2 × 10^−2^
Cell aging	NDAS	4.2 × 10^−3^
Nitric oxide production	4.0 × 10^−2^	5.4 × 10^−3^
Response to nutrients	2.8 × 10^−2^	7.8 × 10^−3^
Endothelial cell, vascular development, VEGFR activity	2.4 × 10^−2^	5.7 × 10^−3^
Cholesterol, lipoprotein for lipids transport	3.5 × 10^−4^	1.5 × 10^−3^
Golgi transport complex, vesicles (endosome, coated, endocytic)	NDAS	9.5 × 10^−3^
Response to DNA damage	6.0 × 10^−3^	1.1 × 10^−3^
Response to steroids	3.0 × 10^−3^	1.3 × 10^−2^
Mineralocorticoid response	9.1 × 10^−2^	1.5 × 10^−4^
Acute inflammatory response	1.79 × 10^−1^	1.7 × 10^−4^
Leukocyte chemotaxis, response to LPS, defense against virus	1.1 × 10^−1^	5.5 × 10^−3^
Monocyte activation	NDAS	5.2 × 10^−2^
**CYTOKINES**		
TGFbeta production	1.1 × 10^−1^	1.0 × 10^−5^
Interleukin IL-1	1.1 × 10^−1^	6.1 × 10^−3^
TNFsuperfamily	1.1 × 10^−1^	1.6 × 10^−2^
Interleukin IL-4	2.7 × 10^−1^	3.0 × 10^−4^
Interleukin IL-6	3.6 × 10^−3^	6.1 × 10^−4^
Interleukin IL-8	2.4 × 10^−2^	1.8 × 10^−4^
Interleukin IL-10	9.6 × 10^−2^	6.1 × 10^−3^
IFNG production	2.5 × 10^−2^	1.0 × 10^−3^
Interleukin IL-18	NDAS	5.2 × 10^−2^
Interleukin IL-23	3.4 × 10^−2^	6.1 × 10^−3^

*Functionalities are derived by ontology analysis of the compatible P-P networks computed on the basis of those corresponding to the nucleus 1, which comes from the whole list of age-dependent genes (for details see the Table S3)*.

* The hypergeometric probabilities are values corrected for the multi-comparisons. In addition, the values indicated are the averages of the probabilities along the several ontology categories that make up each sub-group defined, but weighed according to the relative frequency of the initial ontology classes that resulted from the analysis;

** *NDAS, no-detected as significant*.

From the probability values in Table 2, we conclude that apoptosis in the Younger Group could be two times more frequent than in the Older Group, while the response to steroids could be four times higher. The former is consistent with the high resilience of the Younger Group to accumulate dysregulations leading to neurodegeneration. The second is interesting because the detailed analysis of the steroid response in the Younger Group showed that it facilitates crucial developmental aspects of the nervous system (GATA3, SSTR3, ALMS1, NR2F6, CTNNA1, NCOA, UBE3A, APOB, RARA; *p* = 1.9 × 10^−3^), together with enhanced cholesterol homeostasis and transport (NR5A, ALMS1, APOC2, ABSG5, G6PC; *p* = 2.6 × 10^−6^).

Instead, the response to steroids in the Older Group includes the retinoic acid receptor signaling (RARA/B/G, RXRA/G; 1.5x10^−5^), neurogenesis (FOXA1, NOTCH1, PTCH1, TGFBR1, TGFB2, NR2F2, RARB, MAP1B, IL6, NTRK3; *p* = 3.4 × 10^−3^), cell adhesion (TNF, TGFB2, THBS1, STAT5B; *p* = 4.2 × 10^−4^), regulation of cell stress (F7, IL6, TNF, NTF3, EDN1, THBS1, PPAR, CASP9, PIAS1, SOCS1, UBR5, KDM1A; *p* = 1.86 × 10^−2^), the production of oxygen reactive species (TNF, IL6, EDN1, CDKN1A; *p* = 3.5 × 10^−2^), and the regulation of lymphocyte differentiation with further activation (IL6, RARA, MMP14, STAT5B, IHH, FAS, PDE5A; *p* = 1.5 × 10^−2^).

The regulation of stress proteins in the Older Group is consistent with other distinctive features that we detected in this group: the enhanced transcription of inflammatory cytokines due to the immunological processes (TNF, IFNG, IL18), and the responses to several types of limiting conditions (coagulation disorders, vascular damage, hypoxia, nutrient deficits, DNA damage). In turn, these lead to an increased production of the anti-inflammatory chemokine TGFB (Doyle et al., 2010; Cekanaviciute et al., 2014), nitric oxide production, VEGF production, monocyte activation, increased leukocyte chemotaxis, and axogenesis (see Table 2). Note that the enhanced transcription of retinoic acid receptor activity in the Older Group, could respond to the same need for the observed compensatory axogenesis (see Table 2), by positively influencing myelination (Latasa et al., 2010). Interestingly, some kainate receptors appear up-regulated in the Older Group (GRIK4, GRIK2, GRIA2; *p* = 1 × 10^−3^), although their functional impact is imprecise. This might be due to their opposite roles depending on the pre- or post-synaptic localization, which cannot be established. From Table 2, the deficit in the Older Group of Laminin 5 and Laminin 2, two components of the lamina propria that avoid the breakdown of the blood-brain barrier (Menezes et al., 2014; Yao et al., 2014) is also evident.

We also conclude that transcription of the genes related to the androgen receptor activity in the Younger Group (AR, MED16/13, HDAC6, UBE3A, NCOA2EP300, CCEN1; *p* = 8.7 × 10^−5^) could be 97 times higher than in the Older Group (SMARCA4, PRMT2, CDK7, KAT5, PIAS1, MED14/17; *p* = 8.5 × 10^−3^). In close relation, the genes codifying aromatase activity (CMP2D6, CY2A6, CYP1B1, CYP1A2, CYP19A1; *p* = 5.2 × 10^−7^) appear up-regulated around 1.300 times in the Older Group (see Table S4).

The ontology analysis of the metabolic networks associated to aging confirmed the deficit profile of the Older Group (see Table S4). Hence, the loss of thyroid hormone receptor activity observed there could explain to some extent the lack of an adequate capacity of neurogenesis (Desouza et al., 2005) and the relative loss of neuroprotection (Diez et al., 2008). Glycolysis could be more than 10^4^ times less than in the Younger Group, while the functionalities related with heme/iron binding are 11 times less. Moreover, the following deficits in functions of the secretory apparatus are observed: those related with the stacking of proteins in Golgi (are 9 times less and INPP5E, FUT1, CANT1, B3GALT6 are lacking); the pre-assembly of GPI-anchored protein in the endoplasmic reticulum (is 8.5 times less, and PIGW, PIGL are lacking); the glycosyl transferase activity in Golgi (is 50% of that observed in the Younger Group, and UPP2, ST6GALNAC5, ST3GAL2, PPAT, PIGC, GALNT14, FUT1, B4GALNT1, B3GNT1, B3GALT6, ALG2 are lacking). These findings could be relevant as cause of reticulum endoplasmic stress.

However, from Table S4 we also conclude that Ca^+2^ transport and Ca^+2^-dependent adenylate cyclase (SLC8A1, SLC24A1/A2, ATP2B1/2/3, ADCY1/3) could be increased 2000 times with respect to the expression observed in the Younger Group. Notably, the Older Group showed a response to xenobiotics 1.4 × 10^4^ times higher than the one in the Younger Group. Other important functions related with the age-dependent genes are discussed along the Sections Impact of Sexual Dimorphism on Aging; Impact of Sexual Dimorphism and Aging on Senescence; Impact of Sexual Dimorphism and Aging on Mitochondrial Function; Impact of Sexual Dimorphism and Aging on Autophagy; Impact of Sexual Dimorphism and Aging on MicroRNAs.

#### Effect of sex (age-independent)

The number of probes detected as up-regulated in the Women Group is in relation 2:1 with respect to the ones in the Men Group (Section Factorial Analysis). This quantitative asymmetry supports important dissimilarities in the physiology of the hippocampus due to the sex effects (see Table 3 and Tables S6–S8). Note that if the Sex Ratio^#^ in the corresponding tables is significantly higher than the unity, this means that the transcription of the genes involved is up-regulated in the Women Group (or diminished in the Men Group). On the other hand, when the Sex Ratio^#^ is significantly less than the unity, this means that the transcription of the genes involved is up-regulated in the Men Group (or down-regulated in Women Group).

**Table 3:** **Differential ontology classes at the protein-protein (PP) level for the sex-dependent genes**.

**Ontology process (PP)**	**genes with sex ratio^#^ < 1**	**genes with sex ratio^#^ >1**
	Hypergeometric probability^*^	Hypergeometric probability^*^
Anti-apoptotic mechanisms	8.2 × 10^−3^	6.6 × 10^−11^
Apoptotic mechanisms	2.8 × 10^−2^	3.6 × 10^−4^
Blood coagulation, platelet activation	1.6 × 10^−5^	6.6 × 10^−11^
NGFR signaling	3.5 × 10^−4^	1.6 × 10^−8^
FGFR signaling	4.0 × 10^−4^	4.4 × 10^−6^
Thyroid hormone receptor	4.6 × 10^−3^	1.2 × 10^−6^
Smoothened signaling	1.9 × 10^−1^	4.1 × 10^−3^
Superoxide formation/remotion	8.9 × 10^−2^	4.0 × 10^−3^
SWI/SNF complex, WINAC complex	1.2 × 10^−6^	1.6 × 10^−1^
Axogenesis, axon guidance	1.1 × 10^−4^	6.3 × 10^−5^
Schwann cell proliferation and differentiation	NDAS^**^	1.0 × 10^−3^
Pyramidal neuron development and differentiation	NDAS	1.1 × 10^−3^
Response to hypoxia	7.7 × 10^−2^	3.0 × 10^−4^
Response to insulin	4.3 × 10^−3^	2.5 × 10^−4^
mRNA processing and stability	1.4 × 10^−2^	2.0 × 10^−3^
mRNA splicing	1.9 × 10^−2^	3.6 × 10^−2^
mRNA silencing	NDAS	8.5 × 10^−3^
Steroid hormone signaling	1.6 × 10^−3^	1.48 × 10^−2^
Androgen receptor signaling	6.4 × 10^−3^	1.6 × 10^−1^
Complement activation, C3 membrane attack	1.6 × 10^−5^	7.4 × 10^−1^
Wnt receptor	2.9 × 10^−3^	2.2 × 10^−2^
beta-catenin-APC complex	2.3 × 10^−4^	NDAS
Non-canonical Wnt signaling	1.3 × 10^−1^	3.8 × 10^−2^
TGFbeta signaling	6.3 × 10^−3^	1.7 × 10^−4^
IL1-alpha	NDAS	3.5 × 10^−3^
IL-1 beta	4.5 × 10^−2^	NDAS
IL-1R antagonist	8.4 × 10^−3^	NDAS
Tumor Necrosis Factor	3.5 × 10^−1^	3.1 × 10^−3^
Mastocytes cytokine production		4.0 × 10^−3^
Endothelial cell migration	3.6 × 10^−2^	8.6 × 10^−6^

*Functionalities are derived by ontology analysis of the compatible P-P networks computed on the basis of the corresponding Nucleus 1 from the whole set of specified genes (for details see the Table S4)*.

* The hypergeometric probabilities are values corrected for the multi-comparisons. In addition, the values indicated are the averages of the probabilities along the several ontology categories that make up each sub-group defined, but weighed according to the relative frequency of the initial ontology classes that resulted from the analysis;

** *NDAS, no-detected as significant*.

Based on the probabilities values computed in Table 3, we conclude that in the Women Group the anti-apoptotic mechanisms prevail with respect to the apoptotic mechanisms (5.4 × 10^6^:1 ratio). In the Men Group, instead, this ratio is only 3:1. Moreover, Table 3 also shows that in the Women Group, the following ontology classes as sex-dependent genes, are prevailing: coagulation (2.5 × 10^5^:1 ratio), NGFR signaling (2.2x10^4^:1 ratio), FGFR signaling (90:1 ratio), thyroid hormone receptor signaling (3.8x10^3^:1 ratio), the smoothened signaling (46:1 ratio). The signaling through the transcription factors enhanced in the Women Group could justify that the genes related with the development and differentiation of pyramidal neurons, axogenesis, the higher proliferation and differentiation of Schwann cells and the intense angiogenesis (4 × 10^3^:1 ratio) also appear up-regulated in this group. Perhaps, this could be a compensatory response to the differential increments in several stress responses (266:1 ratio in hypoxic response; 23:1 ratio in superoxide response; 17:1 ratio in insulin response). The occurrence of the interleukin IL-1A (Simi et al., 2007) together with the TNFSF8 (112:1 ratio, present in activated T and B cells; (An et al., 2009), and cytokines associated to mastocytes in the Women Group indicate an active inflammatory state (Dong et al., 2014). However, these inflammatory markers coexists with an increased transcription of genes related with the anti-inflammatory TGFB (38:1 ratio). These data suggest that chronic inflammation is prevailing in the Women Group.

From the analysis of the metabolic networks associated to sex (see Table S7) is evident that the Women Group has an enhanced transcription of genes related with the generation of Nitric Oxide, the membrane proteins of the endoplasmic reticulum, and protein glycosylation. In addition, we conclude that in the Women Group the transcription of genes related with the synthesis of amino acids (1625:1 ratio), the catabolism of amino acids (227:1 ratio), glycerol-phospholipids (36:1 ratio), steroid metabolism (27:1 ratio), lysosome proteins (3.8:1 ratio), one-carbon metabolism (1.41:1 ratio) are also comparatively up-regulated. Concerning the differential expression of steroids, the Women Group naturally showed up-regulated transcription of genes related with the response to oestrogens, which are not significant in the Men Group (see Table S7). But in addition, the Women Group up-regulates the genes related with the responses to glucocorticoids (158:1 ratio) and mineralocorticoids (2.35:1 ratio). Unexpectedly, the genes related with the androgen receptor signaling appear up-regulated in the Women Group (20:1 ratio). This last finding is however consistent with the observed increment in the transcription of the genes with aromatase activity in the same group (225:1 ratio).

On the contrary, from the Table S7 we also conclude that the following metabolic functionalities are up-regulated in the Men Group: transport of carboxylic acids (87000:1 ratio), the response to xenobiotics (13700:1), the tricarboxylic acid cycle and respiration (7250:1 ratio), gluconeogenesis (6720:1), adenylate cyclase activity (97:1 ratio), cGMP catabolism (3:1 ratio). The response to xenobiotics is enhanced in the Men Group (13700:1 ratio; see Table S7). This occurs mainly through the up-regulation of the P450 cytochromes (CYP2 and CYP3 families, Toselli et al., 2016; see Table S8). However, in the Women Group is enhanced the up-regulation of genes codifying glucuronosyl-transferases (UGT1A1/3/6/7/9/10, *p* = 1.1 × 10^−9^), which have great influence not only as detoxifying mechanism and the uptake of flavonoids at the brain-blood barrier (Jäger and Saaby, 2011; Vauzour, 2014), but also conditioning the neuro-steroid balance (Ouzzine et al., 2014). Other important functions related with the sex-dependent genes are discussed along the Sections Impact of Sexual Dimorphism on Aging; Impact of Sexual Dimorphism and Aging on Senescence; Impact of Sexual Dimorphism and Aging on Mitochondrial Function; Impact of Sexual Dimorphism and Aging on Autophagy; Impact of Sexual Dimorphism and Aging on MicroRNAs.

### Validation and predictions

**Figure 4:**
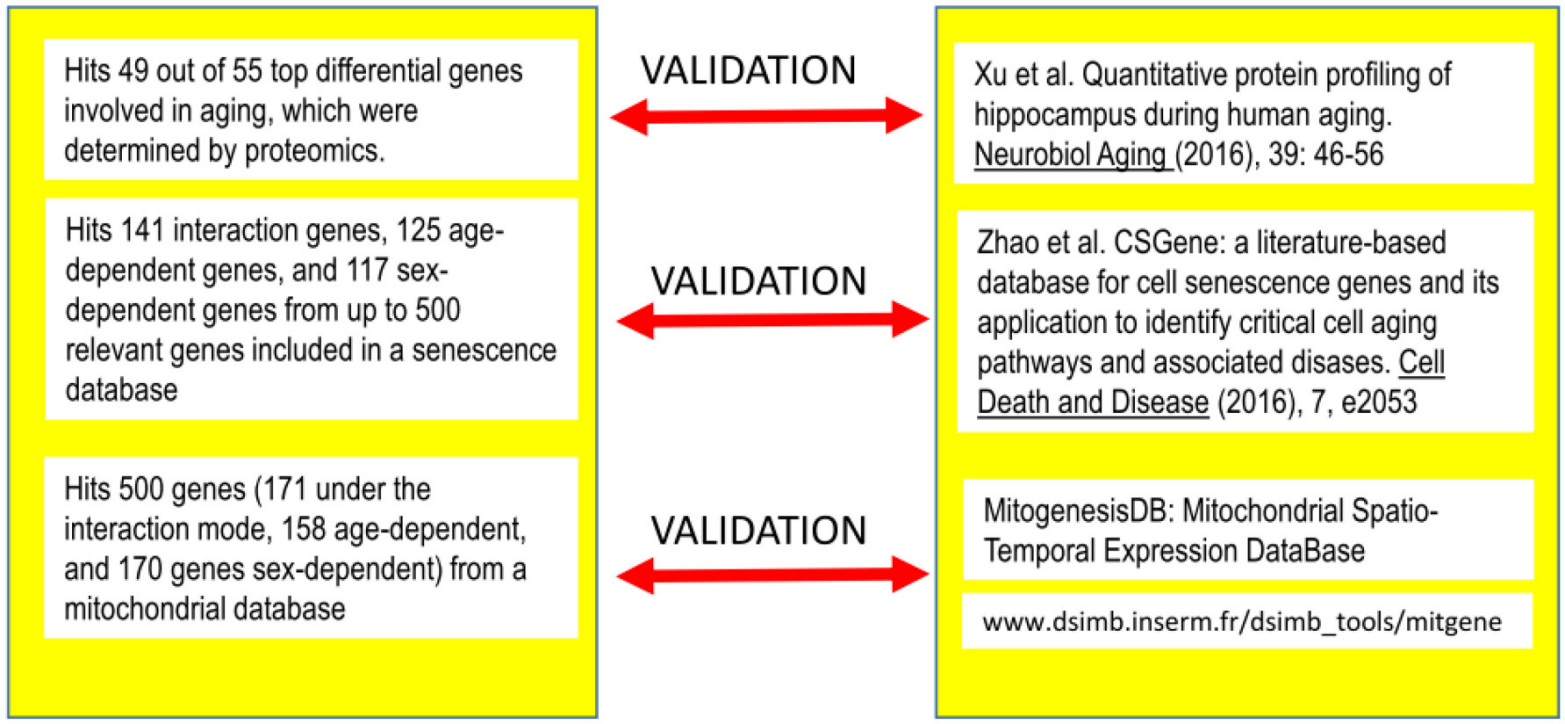
**Correspondence among the genes detected as differentially expressed under the different modes identified after Q-GDEMAR factorial analysis without summarization and the genes identified in three independent sources of data**.

To validate our findings and gain insight into some aspects of the analyses, we compared our results against three external, independent sources of data (see Figure 4).

#### Impact of sexual dimorphism on aging

When we compared the results from factorial Q-GDEMAR with the data reported by Xu et al. (2016), we obtained an excellent concordance. Our approach detected 49 out of 55 top genes (i.e., 90%) found by these authors. For the detail of the genes identified in this comparison see Table S9 (Supplementary Materials).

Data of Xu et al. (2016) were selected for the comparison because this study was designed to analyse genes involved in aging by using proteomics, which can be considered as a reference method (Fu et al., 2009). However, proteomic technique can only detect a maximum of around 4500 proteins as maximum. Microarrays, although more affected by cross-interferences, allow monitoring the complete genome (≈25000 genes). Due to these general features of the microarrays (whole genome mapping) and the particular features of the Q-GDEMAR approach (high sensitivity and low FDR), the genes recognized by Xu et al. (2016) are actually only a sub-set of the genes identified in our study (see Figure 2 and Table S1).

The agreement observed is remarkable for several reasons: First, because often mRNA variations and changes observed at the protein level appear dissociated (Haider and Pal, 2013). Second, because both the analytical methods and the populations sampled were different. In fact, the proteomic study of Xu et al. (2016) sampled Chinese individuals, whereas the microarray study analyzed herein was performed on a Caucasian population. So, we conclude that in this case, it seems that ethnic factors are not very relevant.

#### Impact of sexual dimorphism and aging on senescence

**Figure 5:**
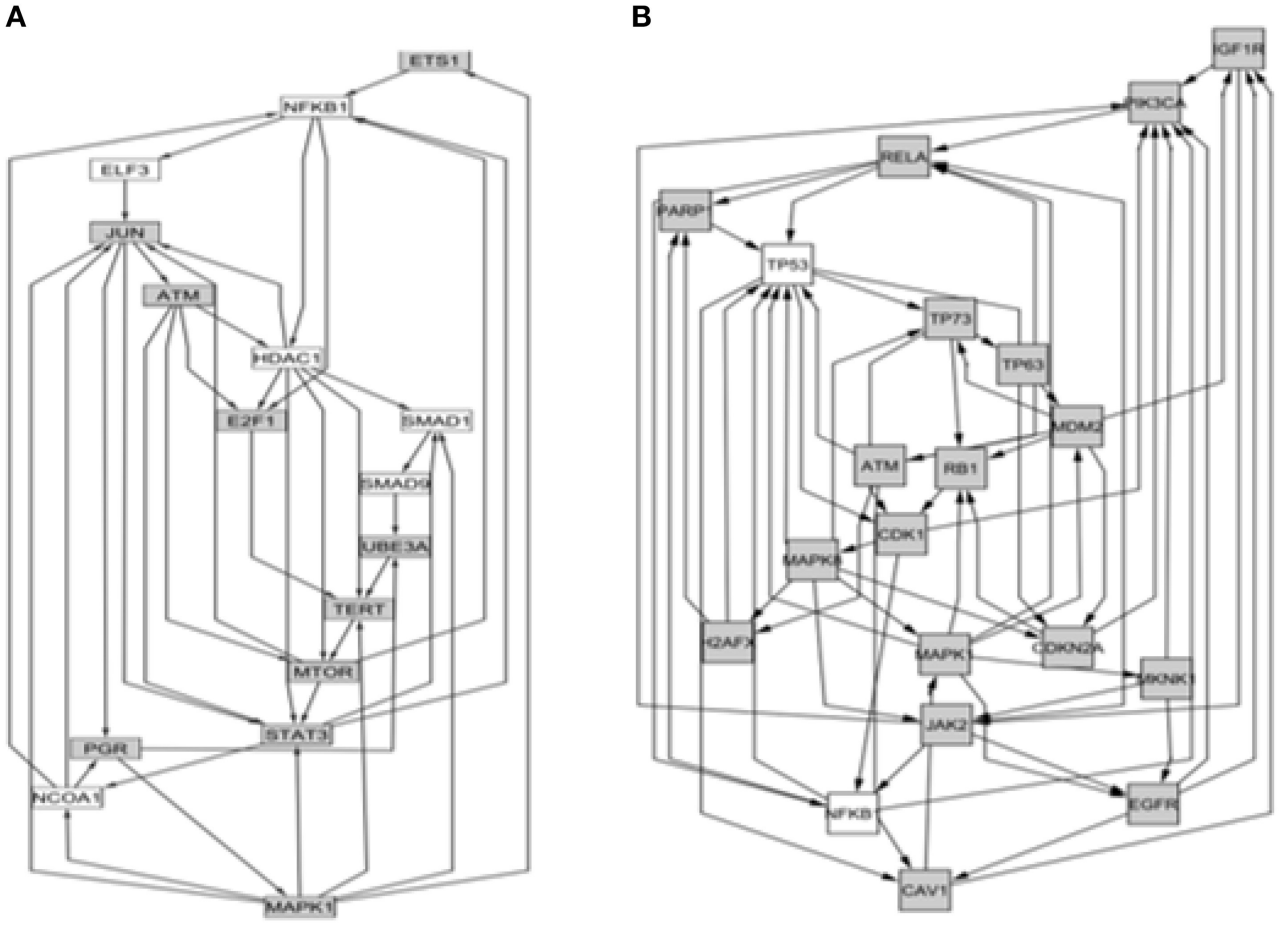
**Comparison between the core of circuits leading to senescence depending on the type of interaction. (A)** Network arising from the nodes with negative value of super-ratio coefficient. **(B)** Network arising from the nodes with positive value of super-ratio coefficient. The nodes fulfilling the condition imposed (sign of the significant super-ratio values) are colored in gray, while colorless nodes represent genes not detected as differentially expressed. These have been added by the computing algorithm for the sake of network completeness because they have well-known interactions with some of the gray nodes.

**Figure 6:**
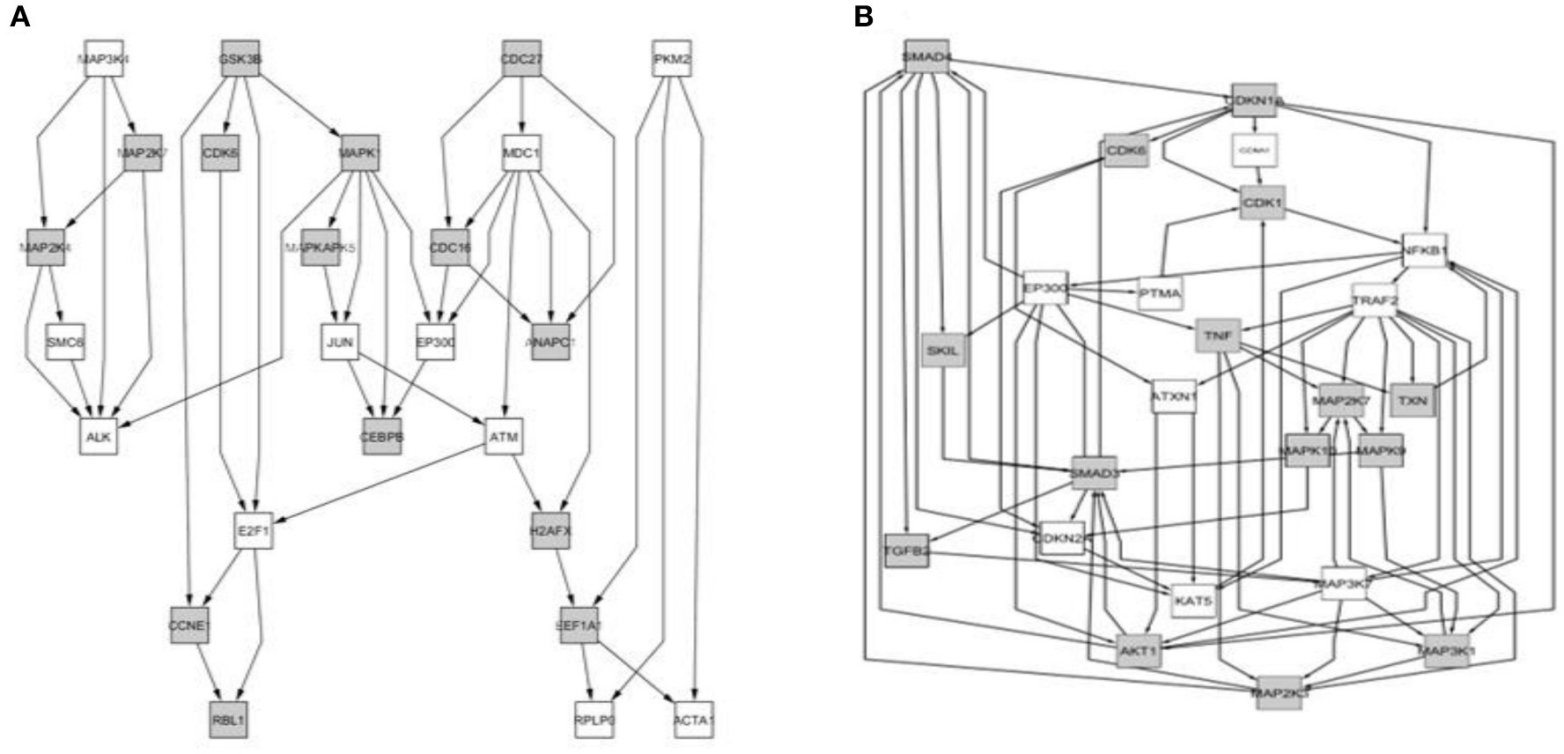
**Comparison between the core of circuits leading to senescence depending on the value of the age effect. (A)** Network of nodes corresponding to samples from the “younger” group; **(B)** Network of nodes corresponding to the samples from the “older” group.

**Figure 7:**
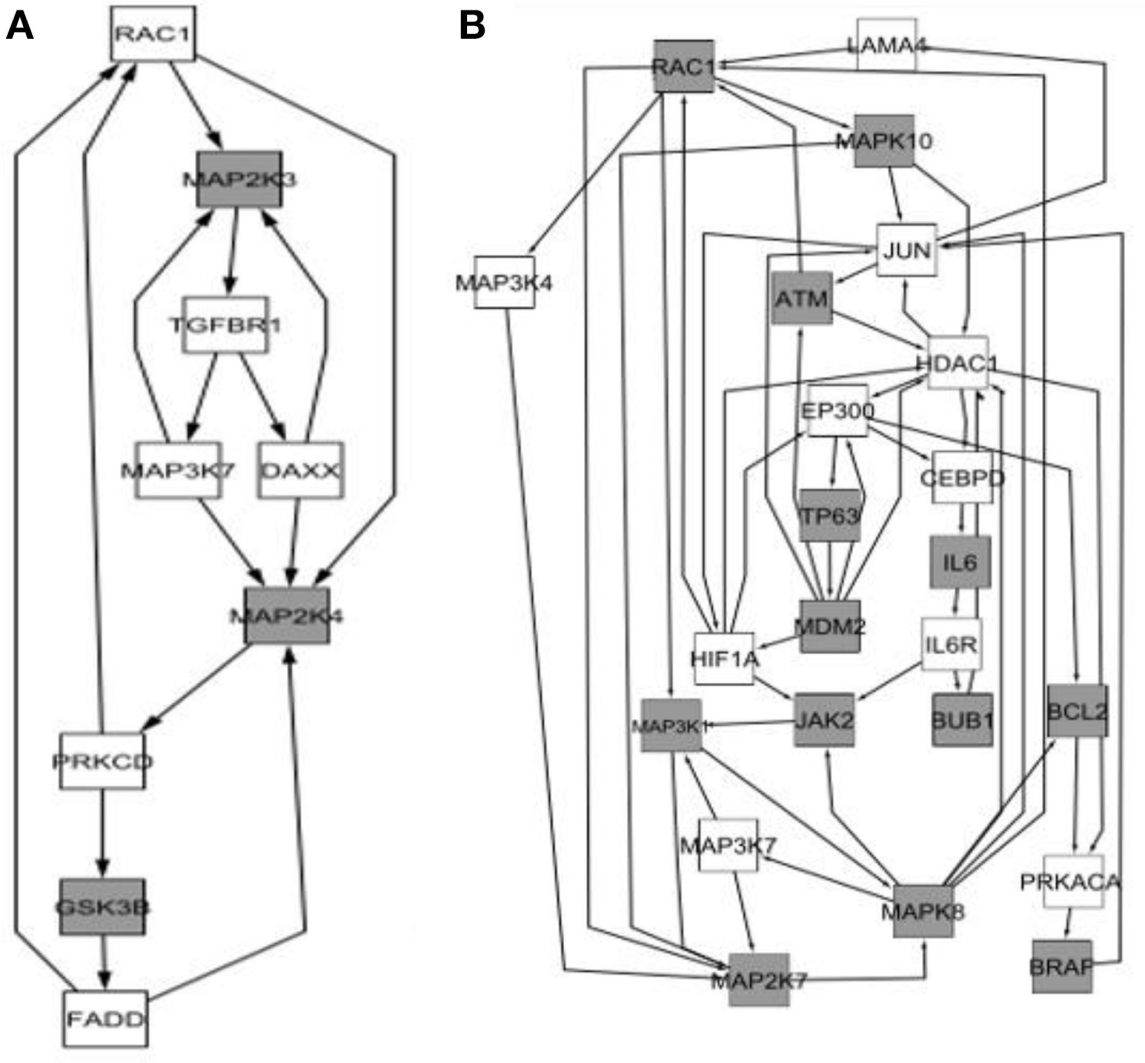
**Comparison between the core of the circuits leading to senescence depending on the sex effect. (A)** Network arising from the genes differentially expressed in the group of “Males”; **(B)** Network arising from the genes differentially expressed in the group of “Females.”

We determined that factorial Q-GDEMAR compared to the senescence database built by Zhao et al. (2016), yielded 141 hits in the case of interaction genes, 125 hits in the case of age-associated genes, and 117 hits in the case of sex-associated genes from a list including 504 relevant genes (see Table S10, Supplementary Materials). Moreover, following the given protocol of network analysis we isolated six sub-networks, which show that senescence mechanisms at the hippocampus involve different mediators depending on age, sex, and the interaction between age and sex (see Figures 5–7).

#### Impact of sexual dimorphism and aging on mitochondrial function

From the comparison of factorial Q-GDEMAR to the MitogenesisDB data (http://www.dsimb.inserm.fr/dsimb_tools/mitgene/) we could identify 500 probes related to mitochondrial functions. Of these, 171 (34.2%) resulted from age-sex interaction, 158 (31.66%) were related to purely age effect, and 170 (34%) were related to purely sex effect. A summary of these findings is presented in Table 4.

**Table 4:** **Main differential features of mitochondrial genes resulting from their age- and/or sex-dependence of several processes in which they are involved (For details see Table S11, Supplementary Materials)**.

**Process**	**Age-sex interaction effect**	**Age effect**	**Sex effect**
Fission	MFF, MTFP1, MTFR2, USP44	NM6, INF2	MTFR1, MTFR2, MIEF1, NME6
Mitochondrial ribosome (small sub-unit)	MRPS24,MRSP21,MRSP12	MRPL42,MRPS22,MRPS17	MRPS22, MRPL42
Mitochondrial ribosome (large-sub-unit)	MRP55,MRPS31,MRPS24, MRPS14, MRPL53, MRPL50, MRPL19, MRPL15	MRLP3, MRPL16	MRPL51, MRPL23, MRL11
tRNA processes	RARS2, HARS2, CARS2	DARS2, EARS2, RARS2, QARS	SARS2, RARS2, EARS2, DARS2, CARS2, QARS
Translation factors	GFM1, TSFM, MTER4	GFM1, TUFM	GFM2, TUFM, MTO
Targeting protein to mitochondria	TOMM34, POLRMT, GFM1, HSP90AA1	TIMM8A	TIMM22, TIMM21, TIMM10B, NFKBIL1,
Mitochondrial carriers	SLC3A2 (neutral, aromatic and branched aliphatic amino-acids)	SLC25A22 (L-Glutamate), SLC1A2 (D-Aspartate), SLC25A19 (Thiamine pyrophosphate), SLC25A24 (glucose, insulin-responsive), SLC25A28 (iron), SLC25A29 (carnitine)	SLC25A32 (Folate carrier), SLC25A31 (Adenin-nucleotides)
Mitochondria organization	TOMM34, POLRMT, GFM1, HSP90AA1	TK2, NOS	TK2, SOD2, GFM2, AIFM2, TP53, JUN, NOS3, NDUSFS8
ATPase coupled H+ movement	ATP5C1, ATP5L, ATP5I,	ATP5C1, ATP5D, ATP5B, ATP5H, ATP5I	ATP5H, ATP5D
Response to ROS	UCP3	UCP2	SOD2
Terminal respiratory chain	UQCRC2	UQCRC2, NDUFB9	NDUFS8, CYCS
Aerobic respiration	ME3, MDH2, IDH2, UQCRC2		MDH2, ACO2, CS
Tricarboxylic acid cycle	MDH2, IDH2	ACO2, CS, IDH1	MDH2, ACO2, CS
Mitochondrial genome maintenance		TK2	TK2, TYMS, TP53
Mitochondrial DNA duplication		TK2, DUT	TK2, JUN, TYMS
Apoptosis mitochondrial DNA changes	APOPT1	CYCS	AIFM2, CYCS, JUN, SOD2
Anti-apoptosis		NME6, NOS3	SOD2, NME6, NOS3
Short-chain fatty acid biosynthesis	HMGCS2	OXSM	HMGCS2

**Figure 8:**
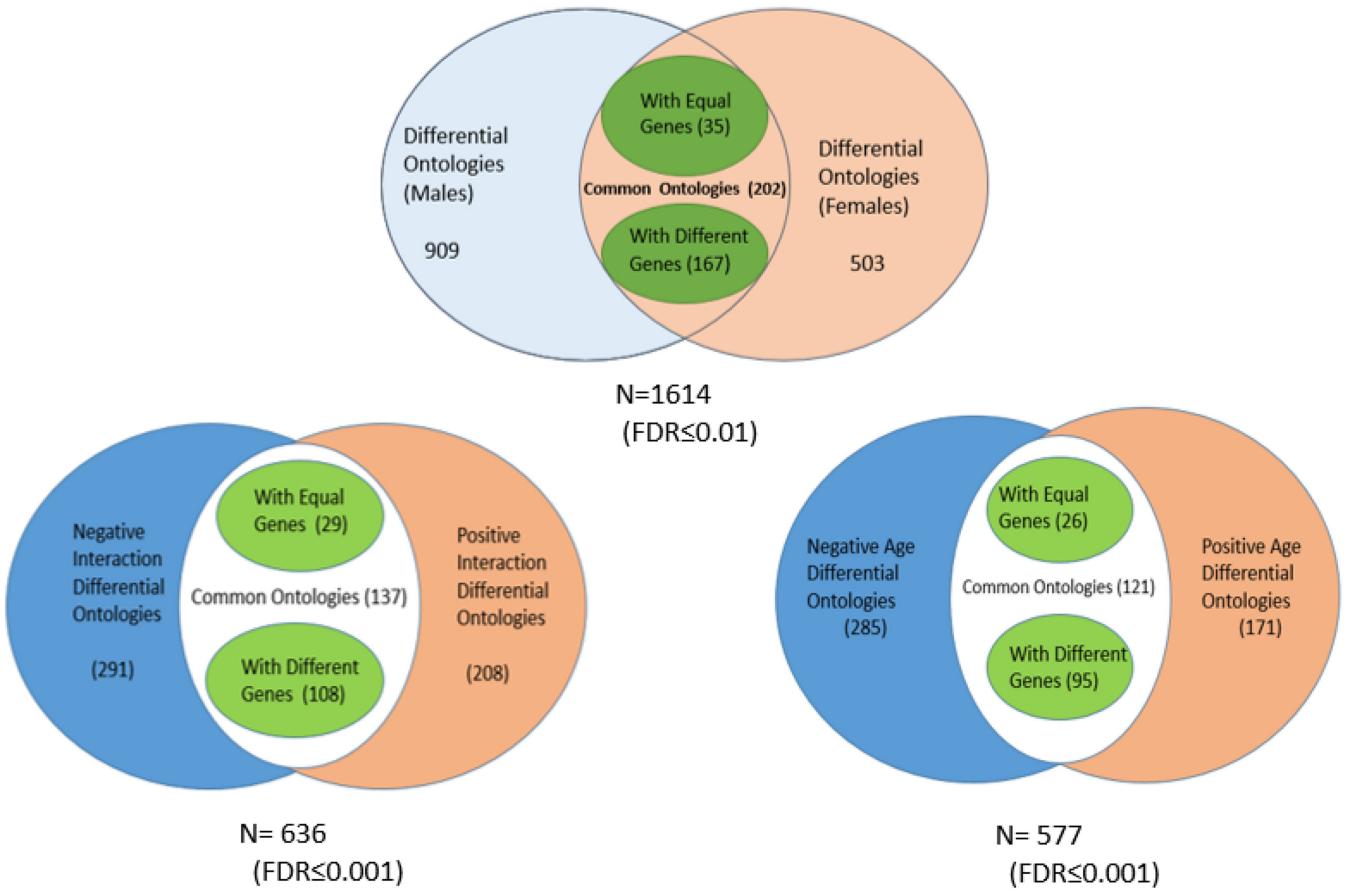
**Distribution of ontology classes in the mitochondrial genes identified by the microarray analysis, according to their association to sex, age, and interaction effects (the values of the FDR associated to each ontology class were corrected for the multiple comparisons)**.

Although the acceptation of some functional differences between the mitochondria coming from aged or younger individuals seems quite natural, the occurrence of mitochondrial differences associated to the sex of the individuals could result less plausible. For the same reasons, it can also be difficult to accept the mitochondrial differences due to the sex-age interaction. However, all these differences can be additionally demonstrated such as is shown in Figure 8.

The results in Figure 8 confirm that the mitochondrial genes from the groups of women and men show dysregulated functionalities that differ significantly. Only 202 out of a total of 1614 significant classes were shared between females and males. While 909 ontology classes were exclusive to males, 503 were exclusive to females. From the sub-set of shared ontology classes, only 35 included the same genes, restricted to a few of monogenic composition (GAR1, POT1, ADH5, and TSG101). Instead, out of the 202 sharing a same functional ontology, 167 use very different sets of genes to accomplish a same biological function. A similar trend is observed in the cases of age and sex-age interactions.

A very important feature of mitochondrial dynamics emerges from the analysis confirming such differences. While a balance between the mechanisms of mitochondrial fusion and mitochondrial fission (van der Bliek et al., 2013) normally exists, the analysis of genes under the interaction mode suggests an enhanced activity of mitochondrial fission in the Older Women Group when compared to the Older Men Group.

In fact, Optical Atrophy 3 (OPA3) was the unique gene we found involved in mitochondrial fusion that was differentially expressed (sex effect = +1.21). This means that the balance between fusion and fission is biased to the latter. This could be an important finding, since mitochondrial fission is frequently observed in Alzheimer's disease (Manczak and Reddy, 2012). Moreover, the claim about fission is also supported by the fact that Mitochondrial Fission Factor (MFF) and Mitochondrial Fission Protein 1 (MTFP1) appeared both with a super-ratio = +1.63, while the Mitochondrial Fission Regulator 2 (MTFR2) showed a super-ratio = +1.59. This trend is reinforced even more due to the up-regulation of other fission effectors, such as Mitochondrial Fission Regulator 1 (MTFR1, sex effect = +1.19), Mitochondrial Elongation Factor (MIEF1, age and sex effects = +1.18), and Inverted Formin 2 (INF2, age effect = +1.20; Huang et al., 2011).

Within the mitochondrial interaction genes the dysregulation of 3-hydroxy-3-methylglutaryl-CoA synthase (HMGCS2, super-ratio = +3), uncoupling protein 3 (UCP3, super-ratio = +1.85), and apoptogenic 1 (APOPT1, super-ratio = +1.77) are also noticeable. While HMGCS2—induced as response to carbohydrate deprivation—influences the synthesis of cholesterol and steroids, UCP3 can dissipate the H^+^ electrochemical gradient, leading to consumption of ATP instead of its generation. UCP3 is induced in the presence of an excessive load of lipids to be derived to beta-oxidation. Note that APOPT1 can lead to apoptosis. All these molecules are enhanced in the group of Old-Women Group compared to the Old-Men Group.

Due to their biological implications, those genes with negative age effect detected (i.e., genes up-regulated during adulthood but down-regulated in old people) are also important (see Table S1, Supplementary Materials). For example, based on the studies performed by Saada et al. (2007), the deficit in the mitochondrial ribosomal protein MRPS22 (age effect = −1.35) would be expected to produce a fall of up to 30% in the activity of Respiratory Complexes I, III, IV, and V. This is a factor of vulnerability because dealing with stress phenomena is impaired. Likewise, the behavior of GPD2 and GPTA2 (age effects = −1.34) might lead to limitations in the synthesis of glycolipids, and hence, the normal composition and biophysical properties of the membranes could be affected. Other detected deficits in the Healthy-Older Group were the mitochondrial carrier for iron SLC25A28 (age effect = −1.37), carnitine carrier SLC25A29 (age effect = −1.41), thiamine pyrophosphate carrier SLC25A19 (age effect = −1.66). All these alterations can negatively impact on normal cell functioning (Nałecz and Nałecz, 1996; Horowitz and Greenamyreb, 2010; Subramanian et al., 2013).

#### Impact of sexual dimorphism and aging on autophagy

**Figure 9:**
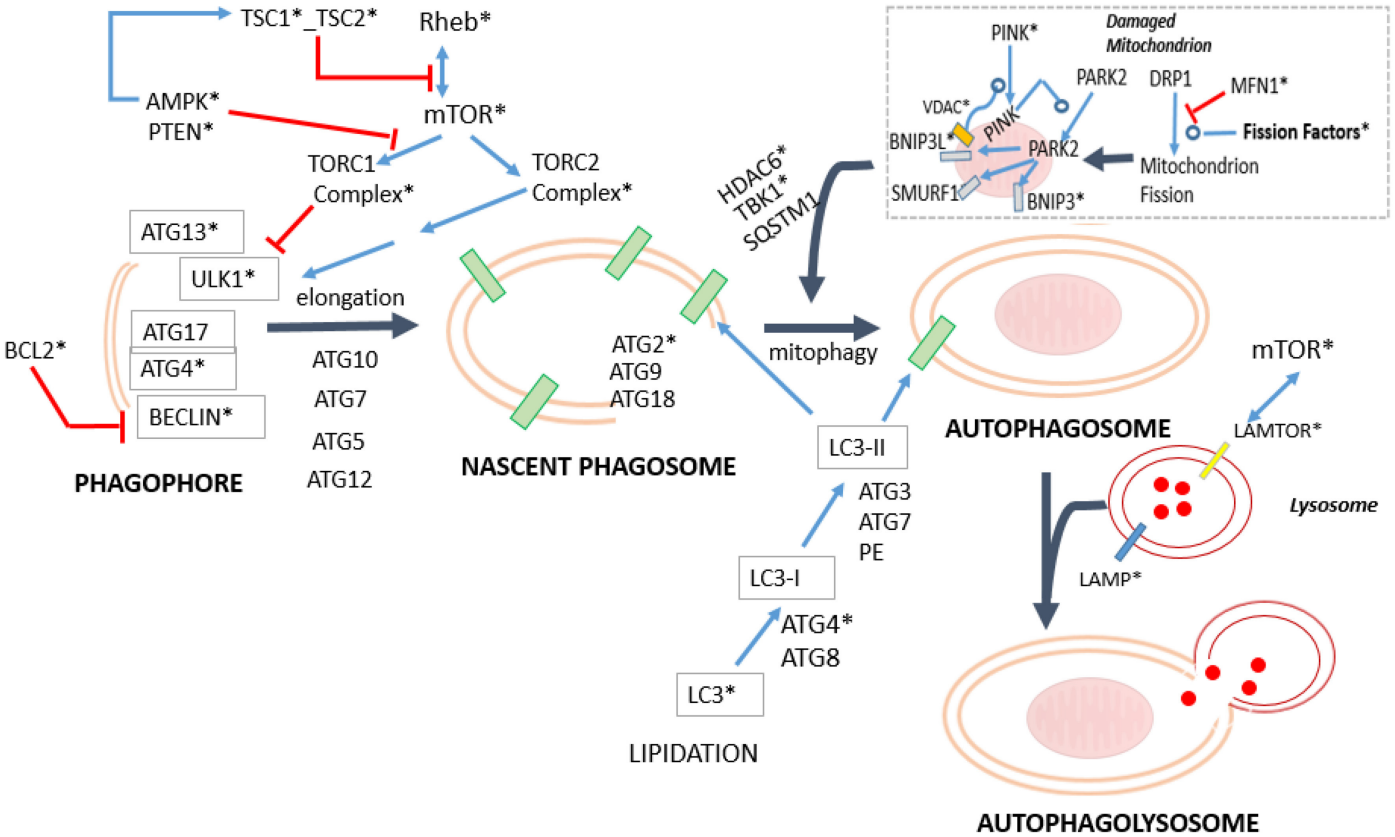
**Simplified diagram mapping the process of mitophagy in relation to the general machinery of autophagy**. The specifically dysregulated genes are marked with asterisk. The blue arrows indicate positive regulation, whereas the red arrows indicate inhibitory effects (For numeric details and disaggregation effects, see Table 5). In mitophagy, there are two complementary mechanisms. One is Parkin-independent, comprising the Drp1 path, (Kageyama et al., 2014) and the RNF185-BNIP1 path (Tang et al., 2011), whereas the other is a group of Parkin-dependent variants: the PARK2-BINP3-NRB1 path (Lee et al., 2011; Pratt and Annabi, 2014), PARK2-BINP3L path (Gao et al., 2015), PARK2-SMURF1 path (Orvedahl et al., 2011). This last path is also used to degrade viral capsids. Actually, the Parkin-independent process is driven by the Dynein-related Protein 1 (Drp1) and regulated through the process of mitochondrial fission (see Section Impact of Sexual Dimorphism and Aging on Mitochondrial Function). It aims to diminish the size of the damaged mitochondria to facilitate their engulfment. Importantly, the PARKIN-dependent mitophagy variants require of the proteins PINK1 (Vives-Bauza et al., 2010), and some of the VDACs isoforms (Sun et al., 2012) to be effective. Both proteins contribute to the recruitment of PARK2 from the cytoplasm to the mitochondria. The mitochondrial PARK2 acts as E3Ub ligase on several adaptor molecules, triggering mitophagy. In addition, note that where PARKIN2 route is used, a final set of proteins such as HDAC6 (Lee et al., 2010) and TBK1 together with SQSTML1 (Matsumoto et al., 2015), which allows the step of engulfment of the mitochondrion.

The identification of some differential features related with apoptosis and the type of the immunological response in the interactions analyses (Section Age and Sex Interaction Effects) and other related with fission in the mitochondrial analysis (Section Impact of Sexual Dimorphism and Aging on Mitochondrial Function) lead us to investigate whether there is any relation between these factors and the mechanisms of autophagy in general and with mitophagia in particular (Ding and Yin, 2012; Kaur and Debnath, 2015). The results obtained are shown in Table 5, and conceptualized in the Figure 9.

**Table 5:** **Disaggregation of effects of sex, age, and sex-age interaction on autophagy and its regulators**.

	**Interaction**	**Sex**	**Age**
**TORC1 AND TORC2 COMPLEXES**			
Rheb	+3.53	+1.29	+1.68
MTOR	−1.73		
RPTOR	+1.80		
AKT1S1 (PRAS40)			−1.40
TTI2	+1.56		
DEPTOR		+1.64	−1.83//+1.30
RICTOR		+1.27//+1.36	−1.37
MAPKAP1 (mSIN1)			+1.24
PTEN	+2.1		
TSC1			+1.19
TSC2		+1.21	+1.25
PRKAA1 (AMPK)		−1.52	
PRKAA2 (AMPK)	+2		
**AUTOPHAGIA (XENOPHAGIA, MICROPHAGIA)**			
ULK1 (ATG1)		−1.31	
ATG13			+1.61
ATG2A		+1.20	
ATG2B	+1.46		+1.19
ATG4C			+1.45
ATG4D		+1.24	
BECN1(ATG6)		+1.29	
BCL2	−1.86	+1.39	
CALCOCO2 (NDP52)		−1.51	
MAP1LC3B1 (LC3)	−1.69		
MAP1LC3B2 (LC3)	−2		
LAMTOR3			+1.55
LAMTOR2		+1.20	
LAMP1			+1.48
LAMP2	−2.30		
LAMP5			−1.33
**MITOPHAGY**			
PTEN	+2.1		
PINK1			−2.65
PARK2			+2.1
SMURF1	−1.88		
HSPB8	−2.63		
MFN1 (Mitofusin 1)		−1.27	
VDAC1		+1.28	
VDAC3	+1.93//+1.79		
BNIP3L			+1.18
BNIP1		+1.18	
RNF185		+1.54	+1.74
MAP1LC3A (LC3)	−1.69		
MAP1LC3B2(LC3)	−2		
TBK1 (TANK-binding Kinase 1)			−2.75
HDAC6	−1.94		−1.52

Importantly, our analysis suggests that mitochondrial fission in the Older-Women Group could not progress to complete the mitophagy process. In fact, the critical effector required PINK1 (Lazarou et al., 2015), appeared with an age-effect = –2.65. This means that its activity is present during adulthood but is lost in the older people. The deacetylase HDAC6 that is required for the maturation of autophagosomes and their fusion with lysosomes (Lee et al., 2010), also appears differentially diminished in the Older-Women Group (interaction effect = −1.94, age-effect = −1.52). The same occurs with MAP1LC3B1/B2 (LC3) that is required for the membrane elongation of the phagophore (interaction effect = −1.69//–2; see Figure 9 and Table 5). Noticeably, the mitophagic activity due to the RNF185-BNIP1 path is potentially possible, because it does not require mitochondrial PARKIN, nor PINK1 (Tang et al., 2011). However, mitophagy finally cannot take place because the mature phagosome is not being produced, as MAP1LC3B1/B2 (LC3) are lacking.

A weak mitophagy activity could exist in the Older-Men Group. The pro-phagocytic factors (PTEN, TSC2, and TSC1) are absent in this group, while the anti-phagocytic regulator mTOR is increased (interaction = −1.73). The regulator PINK1 is attenuated (age effect = –2.65), but MAP1LC3B1/B2 (LC3) is increased (interaction effect = −1.69//–2). The low levels of PINK1 could enable some of the Parkin-dependent routes (PARKIN2 age effect = +2.1). In particular, the route through PARK2-BINP3L (Gao et al., 2015) allows to overcome the anti-phagocytic effect of BCL2, which is strongly increased in the Older-Men Group (interaction effect = −1.86). Interestingly, BINP3L is induced by hypoxia (Bellot et al., 2009), being this condition present in the analyzed group (see Table 1).

As can be seen in Figure 9, mitophagy processes are embedded within a larger basal network aimed to deal with other phagic activies. Thus, in addition to the basal machinery displayed, xenophagy (i.e., the processes for the degradation of the intracellular microorganisms) is mediated by the proteins CALCOCO2 followed by SQTML1 (Xie et al., 2015) and Optineurin (Wild et al., 2011). Instead, the aggresomes (i.e., the degradation process of denatured proteins by phagolysosomes) requires of Hspb8 together with BAG3 (Behl, 2011) or Optineurin (Korac et al., 2013).

Concerning xenophagy, CALCOCO2 was increased in the Men Group (sex effect = −1.51), while SQTML1 and Optineurin were not dysregulated. These data are compatible with a situation in which xenophagy could be more active in the Men Group. However, because BLC2 is strongly increased in the Older-Men Group (interaction effect = −1.86), it inhibits the autophagy initiator BECLIN (see Figure 9). Hence, the apparent advantage in xenophagia due to the CALCOCO2 increment in the Men Group finally results counterbalanced.

Concerning the autophagy of aggresomes, the Hspb8 mediator is increased in the Older-Men Group (interaction effect = −2.98). The mediator BAG3 does not show a differential expression, but BAG2 with similar properties to BAG3, was also increased in the Older-Group (age effect = +1.20). Therefore, it seems that aggresomes are more actively degraded in the Older-Men Group. However, the fact that the anti-phagocytic molecules BCL2 is increased in the Older-Men Group, it finally exerts a counterbalancing effect as with xenophagy.

Note that the expression of BCL2 does not affect the initiation of the “incomplete” mitophagic activity by BECLIN in the Older-Women Group because the BCL2 increment only occurs in the Older Men Group.

#### Impact of sexual dimorphism and aging on MicroRNAs

Although the Affymetrix HG133 plus2 microarray analyzed herein has not been conceived to detect microRNA as its primary objective, the application of our approach by factorial Q-GDEMAR allowed us to detect several dysregulated microRNA genes previously not reported. Interestingly, these microRNAs also can be disaggregated according to the three types of dependence that previously we have defined (see Table 6).

**Table 6:** **Classification of the microRNAs detected as differentially expressed, according to the type of associated effect (Interaction, age, sex)**.

	**Value**	**Targets**	**References**
**INTERACTION EFFECT**			
miR-205	+2.44	CDH1, ZEB1/2, ERRB3, AKT	Carraway et al., 1997
miR-155	+1.90	Inhibits negative regulators of inflammation (SHIP1, SOCS)	Elton et al., 2013
miR-10b	+1.48	HOXA1 and NFKB	Fang et al., 2010; Tonja, 2012
miR-31	+1.38	ICAM1, E-Selectin	Suárez et al., 2010
miR-181	−1.60	Zeb2, MCL1, BCL2L11, BCL2,PTEN, DUSP6, PTPN11	Pati et al., 2014
miR-17	−1.83	APP, TGFBRII, SMAD2, SMAD4, p21, BIM (BCL2L11), PTEN.	Mogilyansky and Rigoutsos, 2013
miR-137 (1558034_s_at)	−7.09	CSMD1, C10orf26, CACNAC1, TCF4, CPLX1, NSF, SYN3, SYT1, BMP6, TGFB2, BAG3, GRIA1 (AMPA), KDM4	Smrt et al., 2010; Collins et al., 2014; Olde Loohuis et al., 2015; Neault et al., 2016
**AGE EFFECT**			
miR-4458	1.82	Hexokinase	Qin et al., 2016
miR-4697 (227084_at)	1.54	Risk for Parkinson by SNP, ERBB2	Persson et al., 2011
miR-99a (231832_at)	1.36	CDK6, Cyclin D	Tao et al., 2015
miR-4435	+1.27	474 predicted targets	
miR-143	+1.18	KRAS, NRAS (neuroblast RAST), ERK, PI3K/AKT, p65 (NFKB), PRKCE, MAPK7 (ERK5), PDGFRA	Pati et al., 2014
miR-Let7b	1.18	CCND (Cycline D) and c-Myc	NCBI gene
miR-Let7d	−1.36	BDNF	Giannotti et al., 2014
miR-137 (1558028_x_at)	−1.36	Risk of schizophrenia by SNP, CSMD1, C10orf26, CACNAC1, TCF4, CPLX1, NSF, SYN3, SYT1, BMP6, TGFB2, BAG3, GRIA1 (AMPA), axonal guidance, EPH receptor signaling, LTP	Smrt et al., 2010; Olde Loohuis et al., 2015
miR-4435	−1.53	No data but present in B cell lymphocytes	Jima et al., 2010
miR-600	−2.07	236 predicted targets	
miR-124-2 (212927_at)	1.47	BDNF, CREB1, Nurr7	Giannotti et al., 2014
**SEX EFFECT**			
miR-99a (231832_at)	1.60	CDK6, Cyclin D	Tao et al., 2015
miR-663a	1.46	CDK6, FBXL18	Shu et al., 2015
miR-4697 (225169_at)	1.33	Parkinson risk, ERBB2	Persson et al., 2011; Nalls et al., 2014
miR-4435 (227674_at)	1.56	Present in B cell lymphocytes	Jima et al., 2010
miR-4435 (221519_at)	1.27	Present in B cell lymphocytes	Jima et al., 2010
miR-4435 (1560119_at)	−1.32	Present in B cell lymphocytes	Jima et al., 2010
miR-124-2 (230021_at)	−1.27	BDNF, CREB1, Nurr77	Giannotti et al., 2014

*When necessary, below the names of the microRNAs, the code of the probe variant for which the effect was observed appears between parentheses*.

Note that, miR-137 is associated to sharp effects in both interaction and age, but actually corresponds to different probes annotated to a same nominal gene. This is in contrast with miR-99a that appears increased in age and sex effects, but is accounted for by the same probe. Finally, it is also notable that miR-4435 showed opposite variations within a same effect (either age or sex), but associated to different probes. Again, some of these biological variations would not have been detected if the classical summarization procedure had been applied.

From a biological point of view, miR-10 and miR-31 showed a significant, convergent effect through their individual positive coefficients under the interaction mode (+1.48 and +1.38, respectively). Both microRNAs act in the endothelial cells, where miR-10 targets HOXA1 and NFKB. As a consequence of HOXA-translation inhibition, E-selectin might result diminished, therefore affecting negatively both, the endothelial sprouting (Tonja, 2012) and the capacity of the endothelial cells to bind neutrophils (Suárez et al., 2010). The increased expression of miR-31 also contributes the same effects. Importantly, although miR-31 is induced by TNF, the occurrence of NFKB inhibition leads to a situation in which the endothelial cells lack the autocrine inflammatory profile (i.e., diminished IL6, IL8, MCP1, VCMA1; Fang et al., 2010). This could be compatible with a situation in which apoptosis of the endothelial cells at the brain microvascular compartment is more probable within the Older Women Group, such as was observed in Table 1.

The negative super-ratio of miR-17 under the interaction mode (interaction = −1.83) is compatible with a situation in which neurogenesis might be significantly more intense in the Older Men Group than in the Older Women Group. This conclusion is in agreement with the independent evidence of neurogenesis shown in Table 1. MiR-17 belongs to the cluster miR-17/92 which comprises other five microRNAs (miR-17, miR-18a, miR-19a, miR-20a, miR-19b1, and miR-92c). The members of this cluster are induced by mitogenic factors (c-Myc, N-Myc, E2F1, E2F3; Mogilyansky and Rigoutsos, 2013). But, with the exception of miR-17, we have not detected altered expression of other members of the cluster. We cannot rule out that there is a finer tuning through co-regulators of the induction. It has also been previously reported that members of the cluster miR-17/92 diminish with aging, but in these cases the interaction effect with the sex had not been assessed (Bates et al., 2009; Grillari et al., 2010). In brief, the very low expression of miR-17 detected in the Older Women Group compared to the observed in the Older Men Group would reflect that there is a diminution of mitogenic activity in the former.

The negative super-ratio observed with miR-181 (interaction = −1.60) is also consistent with the miR-17 finding. In fact, miR-181 is induced by LIF, a cytokine which inhibits the proliferation of stem cells, and hence, it inhibits neurogenesis (Pati et al., 2014). One of the target of miR-181 is MCL1, which however, has two isoforms: while the isoform 1 is anti-apoptotic, the isoform 2 is pro-apoptotic. Unfortunately, the HG133plus2 microarray cannot differentiate between the MCL1 isoforms, but we have detected a MCL1 probe (10804_x_at) with high interaction super-ratio (+2.36), whereas other MCL1 probe (1563494_at) appears associated to positive age (+1.67) and negative sex effect (−1.40), respectively. Here lies the importance of being able to discriminate the different isoforms of a given chemical species.

The evaluation of miR-181 effects according to its targets, suggests that Older Men Group could have higher levels than Older Women Group of the following activities: more cell adhesion (i.e., more CdhE due to low ZEB2), viability (more PI3K/AKT due to low PTEN), more MAPK signaling (i.e., potentially more ERK^*^, JUN^*^, p38^*^), more pro-apoptotic activity (less Bcl2), and low cytoskeleton organization (less RhoA activation due to the loss of PTPN11 effect on Rock2).

Concerning the purely aging effects, the detection of slight but significant positive effect of miR-143 (age effect = +1.18) implies the occurrence of IFG1 given that this molecule induces this microRNA. The fact that IGF1 induces TERT (probe 1555168_a_at), which appears with a positive age effect (+1.47), and a positive sex effect (+1.41) also corroborates this implication. Of note is that TERT also occurs with a negative super-ratio in the interaction column (−1.90), but this case was associated to other probe (204172_at). This would indicate that the TERT molecule present in the Older Women Group is not strictly the same as the one present in the Older Men Group. In fact, it is known that TERT has different isoforms with different activities (Rousseau et al., 2016). In any case, the action of IGF1 is important, because in contrast with LIF, IGF1 leads to proliferation and lineage commitment of cell precursors of astrocytes, oligodendroglia and neurons (Pati et al., 2014), while the shortening of telomeres due to lower TERT activity is involved in the phenomenon of replicative senescence (Wei et al., 2015; Pérez-Martín et al., 2016).

Interestingly, miR-137 showed a very high, negative interaction (super-ratio = −7) much higher than that observed for the same microRNA as age effect (coefficient = −1.36). This means that the process of neuritogenesis and the glutamatergic synaptic transmission might be much more inhibited in the Older Men Group than in the Older Women Group. According to the targets of miR-137 which are known, the first process can be mediated by Mib1 down-regulation (Smrt et al., 2010), while the second one is mediated by the down-regulation of GRIA1, a critical component of AMPA receptors (Olde Loohuis et al., 2015).

The highly negative interaction effect detected in miR-137 could have some un-expected effects. As the target molecule CPLX1 (Complexin 1) diminished due the presence of high-miR-137 in the Older Men Group compared to the Older Women Group, an increased release of glutamate from the pre-synaptic vesicles could be expected, because SNARE will be activated (Martin et al., 2011). However, because SYN3 (Synapsin III) will also be diminished (SYN3 is another miR-137 target), the presynaptic vesicles would be unavailable for release because they would not be bound to the cytoskeleton (Tang et al., 2015), thus any observable effect is neutralized. On the contrary, in the Old Women Group, where miR-137 is lower, CPLX1 will be increased inhibiting SNARE, whilst SYN3 will also be increased, favoring the availability of vesicles. One might speculate that for opposite reasons, both the Older Men Group and the Older Women Group could have a null final effect concerning their targets CPLX1 and SYN3 despite their differential content in miR-137.

In the case of miR-600 a very significant diminution appeared associated to purely age effect (−2.07). Although no specific bibliographic data was found, this microRNA could be associated with up to 236 potential targets. Important homeostatic regulators (IRS1, RICTOR, RHEB, PP2ACA, STIM2, ATPVOA2), a negative modulator of Sonic Hedgehog signaling (GLI3), and very important cognitive regulators (FMR1, CCK1), together with other critical molecules (ORC2, CREBBP, ATF3, RAB10, GAD1, Dynein) have been described among them.

Concerning the influence of a purely sex effect, mir-124 appeared with a negative sex coefficient (−1.27). It means that mir-124 activity is slightly but significantly higher in men than in women. Some negative impact on the glutamatergic transmission can be expected, owing to the targets BDNF and CREB that miR-124 regulates.

Other important microRNAs detected as sex-associated were miR-99a and miR-663a. Both share an inhibitory effect on CDK6. In fact, neuronal differentiation after the neurogenesis process could result more inhibited in women compared to men, because of the lack of activation of PAX6 by CDK6 (Shu et al., 2015). The down-regulation of FBXL18 by miR-663A also contributes to this effect. FBXL18 is required to form a complex with SKP1 and CUL1 to allow the degradation of cyclins and consequently exit from the mitotic process. Moreover, based on literature reports it can be expected that the decrease in miR-99a in males exerts a neuroprotective effect, such as that observed under oxidative stress conditions (Tao et al., 2015). This is justified because up to 56 target of miR-99a have been predicted (among others MTOR, EIF2C, FGFR3, BMP2R, KDM6B) from TargetScan database.

Within the purely sex-associated effect, miR-4435 (sex coefficient = +1.56), and miR-4697 (sex coefficient = +1.33) also appeared. The microRNA database predicted the occurrence of 474 potential targets for miR-4435, while in the case of miR-4697 the predicted targets are 106. Taking together these results, it can be speculated that some critical molecules such as Apo2, VLDLR, TGFB2, TGFBR2, CDK6, MAPK8 (JNK), neurotransmitter transporter SLC6A4, ATF3, GPX7, CDC42EP, TSPAN5 (tetraspin 5), TTP3 (tubulin polymerization promoting factor), BAI3 (brain-specific inhibitor of angiogenesis), MAPT (microtubule associated protein Tau), the pro-inflammatory enzyme PTSG2 (COX2), CDK5R1 (p35, regulatory sub-unit), ATM (DNA-damage signaling) might be more diminished in women than in men.

## Concluding remarks

The application of suitable experimental design (factorial analysis) together with an optimized method of microarray analysis (Q-GDEMAR without summarization) presented herein has generated a quarry of data to be mined.

Although the reliability of the results is very high (low enough FDR values were achieved), interpretation of the data is still a challenge. This challenge derives from the intrinsic quantitative complexity of the information (some thousands of significant genes were detected), as well as its great qualitative complexity (there is a high interconnectivity within and between many different classes of functionalities).

Though we are aware of some uncertainties, mainly associated to the fact that we analyzed the transcriptome rather than the proteome, we have presented a practical approach that proves appropriate to deal with the stated problem (see Figure 1). In fact, the comparisons made with other proteomic studies, and other indirect but multiple validations performed are highly satisfactory.

In this work we have privileged the presentation of some of the most general emerging trends. The objective pursued was mainly to demonstrate that sexual differences in the human hippocampus exist, and have biological consequences. Many other differences at different levels remain to be analyzed further. Beyond the particular biological inferences made along the text, it is important to emphasize that as relevant as the sex differences, is the detection of the interactions between sex and age, which should be considered separately from the age and sex pure effects.

A better understanding of the phenomena in the aging brain might allow to design successful therapies for neurodegenerative pathologies. In this context, it appears relevant to clarify whether women and men follow the same path during the transition from healthy aging to neurodegenerative disease stages. To our knowledge, this task has not been previously addressed in the literature.

## Author contributions

DG designed and performed the study. NT supervised the work. DG and NT wrote the manuscript.

### Conflict of interest statement

The authors declare that the research was conducted in the absence of any commercial or financial relationships that could be construed as a potential conflict of interest.

## Supplementary material

Figure S1 **Correlation between interaction coefficient and super-ratio**.

Table S1 **Differential expressed genes identified on micro-array GSE11882 according to Q-GDEMAR factorial approach**.

Table S2 **Ontology analysis on the Protein-Protein networks (Nucleus 1, strongly connected components) according to the interaction effects**.

Table S3 **Ontology analysis on the Protein-Protein networks (Nucleus 1) according to the age-dependent effects**.

Table S4 **Comparison of main functional classes associated to metabolic networks (Nucleus 1) according to the age-dependent effects**.

Table S5 **Ontology analysis on the metabolic networks (Nucleus 1) according to the age-dependent effects**.

Table S6 **Ontology analysis on the Protein-Protein networks (Nucleus 1) according to the sex-dependent effects**.

Table S7 **Comparison of main functional classes associated to the whole metabolic network according to the sex-dependent effects**.

Table S8 **Ontology analysis on the metabolic networks (whole network) according to the sex-dependent effects**.

Table S9 **Comparison between the genes identified by factorial Q-GDEMAR as dysregulated and the top-genes detected by proteomics in the aging study performed by Xu et al. (2016)**.

Table S10 **Comparison between the genes identified by factorial Q-GDEMAR as dysregulated and the genes included in senescence data-base built by Zhao et al. (2016)**.

Table S11 **Detail of the dysregulated genes with mitochondrial activity detected by factorial Q-GDEMAR, stratified according to the type of dependence on age and sex**.
