# Supplementary material for: Sexual Dimorphism and Aging in the Human Hyppocampus: Identification, Validation, and Impact of Differentially Expressed Genes by Factorial Microarray and Network Analysis
Source: Front Aging Neurosci. 2016 Oct 5;8:229. doi: 10.3389/fnagi.2016.00229 (PMC5050216; doi:10.3389/fnagi.2016.00229)
Supplement: Figure S1 — Correlation between interaction coefficient and super-ratio. [file Image1.pdf]

Supplementary Materials

Sexual dimorphism and ageing in the human hyppocampus: Identification, validation and impact of differentially expressed genes by factorial microarray and network analysis

Daniel V. Guebel[1,2] and Néstor V. Torres[2*]

[(1)]Biotechnology Counselling Services. Buenos Aires. Argentina

[(2)]Systems Biology and Mathematical Modelling Group. Departamento de Bioquímica, Microbiología, Biología Celular y Genética. Facultad de Ciencias. Universidad de La Laguna. San Cristóbal de La Laguna. Tenerife. España.

e-mail (DVG): dvguebel@hotmail.com *e-mail (NVT): ntorres@ull.edu.es

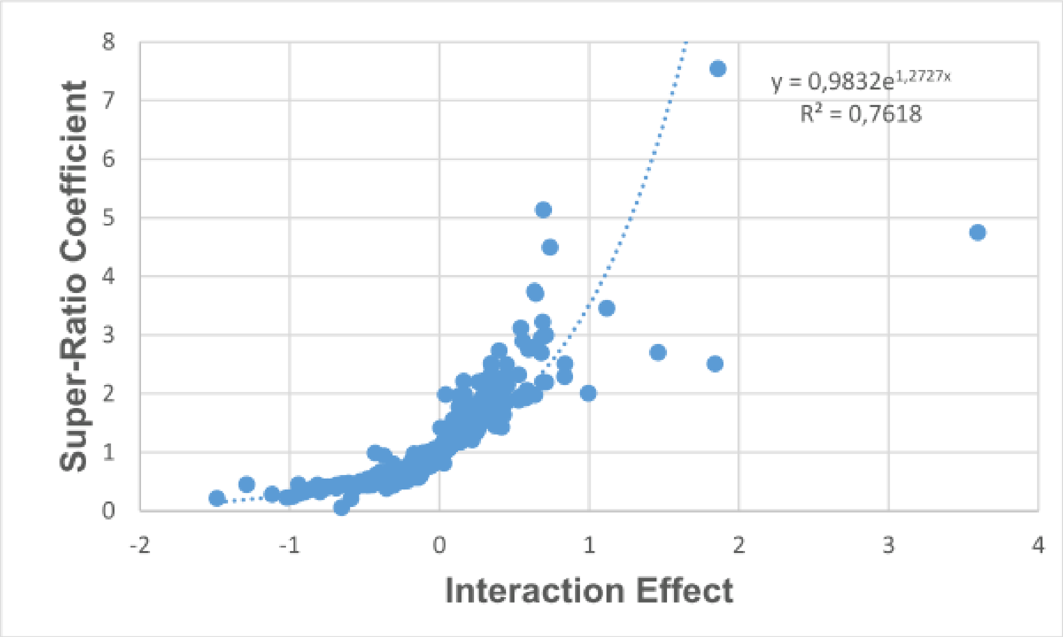

Figure 1S. Correlation between the magnitude of the interaction effect and the super-ratio coefficient (n=2000 probes)
