# Supplementary material for: Sexual Dimorphism and Aging in the Human Hyppocampus: Identification, Validation, and Impact of Differentially Expressed Genes by Factorial Microarray and Network Analysis
Source: Front Aging Neurosci. 2016 Oct 5;8:229. doi: 10.3389/fnagi.2016.00229 (PMC5050216; doi:10.3389/fnagi.2016.00229)
Supplement: Table S4 — Comparison of main functional classes associated to metabolic networks (Nucleus 1) according to the age-dependent effects. [file Table4.pdf]

Supplementary Materials

Sexual dimorphism and ageing in the human hyppocampus: Identification, validation and impact of differentially expressed genes by factorial microarray and network analysis

Daniel V. Guebel[1,2] and Néstor V. Torres[2*]
[(1)]Biotechnology Counselling Services. Buenos Aires. Argentina
[(2)]Systems Biology and Mathematical Modelling Group. Departamento de Bioquímica, Microbiología, Biología Celular y Genética. Facultad de Ciencias. Universidad de La Laguna. San Cristóbal de La Laguna. Tenerife. España.
e-mail (DVG): dvguebel@hotmail.com *e-mail (NVT): ntorres@ull.edu.es

Table S4: Main metabolic functional classes prevailing by the Age Ratio# discriminating criterion. Functionalities are derived by ontology analysis of the compatible metabolic networks built from nucleus 1, derived from the whole list of age-dependent genes (for details see the Supplementary Table S5).

Ontology Process (metabolic)	Set of genes with Age Ratio# < 1	Set of genes with Age Ratio#> 1
	Hypergeometric Probability*	Hypergeometric Probability*
Hexoses metabolism, Glycolysis	1.8×10^{-6}	4×10^{-2}
Mitochondrial matrix, aerobic respiration, respiratory electron chain	7.0×10^{-3}	1.5×10^{-4}
Glycerolipids, Lipid biosynthesis	8.4×10^{-7}	NDAS
Dicarboxylic Acid transport	6.0×10^{-7}	4.5×10^{-9}
Monocarboxylic Acid transport and metabolism	1.8×10^{-6}	3.7×10^{-5}
Glutamine and glutamate transport	NDAS	6.3×10^{-7}
Serine, branched amino-acids, and proline transport;	3.0×10^{-7}	NDAS
Thyroid hormone transport	1.5×10^{-6}	NDAS
Heme /Iron-binding	2.8×10^{-6}	3.1×10^{-5}
Golgi cysterna membrane and Golgi stacking. Microsome	3.0×10^{-5}	2.6×10^{-4}
Glycosyl-transferase activity in Golgi	2.0×10^{-5}	4.2×10^{-5}
Pre-assembly GPI-anchored in ER membrane	6.0×10^{-7}	5.1×10^{-6}
Xenobiotic response	6.4×10^{-8}	4.4×10^{-12}
Nucleosides biosynthesis and nucleotide savage	7.8×10^{-7}	3.7×10^{-7}
3',5'-cyclic –AMP phosphodiesterase activity	5.4×10^{-7}	4.3×10^{-6}
Aromatase activity	6.8×10^{-4}	5.2×10^{-7}
Calcium homeostasis	$5.9 \times 10\text{-}^{2}$	2.7×10^{-5}

* The hypergeometric probabilities are values corrected for the multi-comparisons. In addition, the values indicated are the averages of the probabilities along the several ontology categories that make up each sub-group defined, but weighed according to the relative frequency of the initial ontology classes that resulted from the analysis; ** NDAS: no-detected as significant
