# Supplementary material for: Sexual Dimorphism and Aging in the Human Hyppocampus: Identification, Validation, and Impact of Differentially Expressed Genes by Factorial Microarray and Network Analysis
Source: Front Aging Neurosci. 2016 Oct 5;8:229. doi: 10.3389/fnagi.2016.00229 (PMC5050216; doi:10.3389/fnagi.2016.00229)
Supplement: Table S7 — Comparison of main functional classes associated to the whole metabolic network according to the sex-dependent effects. [file Table7.pdf]

Supplementary Materials

Sexual dimorphism and ageing in the human hyppocampus: Identification, validation and impact of differentially expressed genes by factorial microarray and network analysis

Daniel V. Guebel[1,2] and Néstor V. Torres[2*]

[(1)]Biotechnology Counselling Services. Buenos Aires. Argentina

[(2)]Systems Biology and Mathematical Modelling Group. Departamento de Bioquímica, Microbiología, Biología Celular y Genética. Facultad de Ciencias. Universidad de La Laguna. San Cristóbal de La Laguna. Tenerife. España.

e-mail (DVG): dvguebel@hotmail.com *e-mail (NVT): ntorres@ull.edu.es

Table S7: Differential functional classes associated to metabolism for the set of sex-dependent genes. Functionalities are derived by ontology analysis performed on the whole compatible network (for details see the Supplementary Table S8).

Ontology process (Metabolic)	**genes with Sex Ratio# <1**	**genes with Sex Ratio# >1**
	Hypergeometric probability	Hypergeometric probability
Xenobiotic response, drug response	3.2×10^{-8}	4.4×10^{-6}
Adenylate cyclase	8.0×10^{-6}	7.8×10^{-4}
3',5'-cyclic nucleotide phosphodiesterase	1.1×10^{-5}	3.2×10^{-5}
c-GMP catabolism	6.6×10^{-7}	8.0×10^{-4}
Carboxylic Acid Transport	1.0×10^{-7}	8.7×10^{-3}
Gluconeogenesis, glucose biosynthesis	3.3×10^{-6}	6.3×10^{-3}
Tricarboxylic acid cycle (TCA), respiration	1.2×10^{-6}	8.7×10^{-3}
Amino acid biosynthesis	1.3×10^{-1}	8.0×10^{-5}
Amino acid catabolism	5.9×10^{-2}	2.6×10^{-4}
Glycero-phospholipids	1.3×10^{-2}	3.6×10^{-4}
Phospho-Inositosides metabolism	1.2×10^{-1}	1.3×10^{-4}
One-Carbon metabolism	1.5×10^{-4}	4.1×10^{-4}
Glutathione metabolic processes	8.2×10^{-4}	5.8×10^{-4}
Steroid response	1.3×10^{-2}	4.8×10^{-4}
Aromatase activity	7.2×10^{-3}	3.2×10^{-5}
Response to nitrosative compounds	5.8×10^{-7}	1.6×10^{-5}
Superoxide, hydrogen peroxide	6.2×10^{-3}	1.1×10^{-2}
Nitric Oxide generation and regulation	NDAS**	4.2×10^{-4}
Caffein oxidase	5.8×10^{-7}	6.2×10^{-8}
Endoplasmic reticulum membrane protein	NDAS	2.8×10^{-4}
Glycosylation of proteins	NDAS	1.3×10^{-5}
Golgi membrane proteins	1.7×10^{-1}	8.7×10^{-5}
Lysosome proteins	1.3×10^{-3}	3.4×10^{-4}

* The hypergeometric probabilities are values corrected for the multi-comparisons. In addition, the values indicated are the averages of the probabilities along the several ontology categories that make up each sub-group defined, but weighed according to the relative frequency of the initial ontology classes that resulted from the analysis; ** NDAS: no-detected as significant.
