# Supplementary material for: Sexual Dimorphism and Aging in the Human Hyppocampus: Identification, Validation, and Impact of Differentially Expressed Genes by Factorial Microarray and Network Analysis
Source: Front Aging Neurosci. 2016 Oct 5;8:229. doi: 10.3389/fnagi.2016.00229 (PMC5050216; doi:10.3389/fnagi.2016.00229)
Supplement: Table S9 — Comparison between the genes identified by factorial Q-GDEMAR as dysregulated and the top-genes detected by proteomics in the aging study performed by Xu et al. (2016). [file Table9.pdf]

Supplementary Materials

Sexual dimorphism and ageing in the human hyppocampus: Identification, validation and impact of differentially expressed genes by factorial microarray and network analysis

Daniel V. Guebel[1,2] and Néstor V. Torres[2*]
[(1)]Biotechnology Counselling Services. Buenos Aires. Argentina
[(2)]Systems Biology and Mathematical Modelling Group. Departamento de Bioquímica, Microbiología, Biología Celular y Genética. Facultad de Ciencias. Universidad de La Laguna. San Cristóbal de La Laguna. Tenerife. España.
e-mail (DVG): dvguebel@hotmail.com *e-mail (NVT): ntorres@ull.edu.es

Table S9: Comparison between proteins altered during ageing as detected by proteome analysis (Xu et al., 2016) with respect to our results using factorial Q-GDEMAR (micro-array data from Berchtold et al., 2008). In both the samples were taken from the hippocampus region, but in the proteomic study the samples come from a chinese population, whereas in the case of micro-array the samples come from a caucasian population.

Proteome Analysis	Microarray analysed by factorial Q-GDEMAR		
[Eldered]	Age Effect [Eldered to Adult Ratio]	Sex Effect [Female to Male Ratio]	Sex-Age Interaction [Super-Ratio]
HPCA			HPCA: +2.14
BGN	BGN: +1.20	BGN: -1.43	
TPM1	TMP4: -1.60		TPM2: +1.73<br>TPM3: +1.92
MYH11	MYH2: +1.93<br>MYH3: -1.37<br>MYH8: +1.19<br>MYH10: +1.41		MYH11: +2.04<br>MYH10: +1.20
GSTM4	GSTM5: +1.24	GSTM3 +1.30	
CAPS2			CAPS2:+1.71<br>CAPS2: -2.19
SYNPO2	SYNPO2: +1.57<br>SYNPO: -1.68	SYNPO: -1.37	
VIM			
PMP2			PMP2: +1.87
TAGLN	TAGLN: -1.80	TAGLN: -1.55	
FHL2	FHL1: +2.04<br>FHL3: +1.36<br>FHL5: +1.26		FHL1: +3.18<br>FHL1: -1.64
CAS			CASR: +2.03
FTL			
RIMS3			RIMS4: +1.26
TF (transferrine)			TF: +1.19<br>TFR2: +1.47
BDH2			HIBADH: +9.84
CTSH	CTSB: -1.45<br>CTSC: -1.52<br>CTSF: -1.37		CTSS: +1.96<br>CTSB: +1.89<br>CTSG: +1.59<br>CTSL: -2.28

GPRC5B		GPRC5B: +1.18	
COL6A2	COL6A2: -1.50		COL6A5: +1.46
COL6A3	COL6A1: +1.24	COL6A1: +1.35	COL6A1:+1.55
HLA-Class I	HLA-C: -1.55		HLA_A2: +2.11<br>HLA-B: +1.44<br>HLA-E: -1.68<br>HLA-G:+1.41<br>HLA_A2: +2.11
HLA-Class II	HLA-DPA2: +1.47<br>HLA-DQA1: -1.33<br>HLA-DQA2: -1.33<br>HLA-DRB4: +1.24<br>HLD-DRB5: +1.29	HLA-DRA:-1.26	HLA-DRB1: +1.24<br>HLA-DRB2: +1.14<br>HLA-DRB6: -2.55<br>HLA-DPA1: -3.57<br>HLA-DQA1: -1.41<br>HLA-DQB1: +2.18<br>HLA-DOA: +1.56
RENBP??			
H2AFY	H2FY: -1.38<br>H2APY2: -1.40<br>H2AFX: -1.88		H2AFY: -1.958
ITPKB	ITPKA: -1.32	ITPKB: 1.27	ITPKB: +1.89<br>ITPKB: -1.23
FLNA	FLNC: +1.38		FLNB: +1.68<br>FBLIM1: -1.60
GFAP			
ANXA2		ANXA2: -1.65	ANXA2: +1.54<br>ANXA2: +1.62<br>ANXA2: -2.31
NUDTIC	NUDT2: +1.22<br>NUDT4: +1.24<br>NUDT12: +1.59<br>NUDT13: +1.20<br>NUDT22: +1.43<br>NUDC: 1.19<br>NUDT16R1: 1.19	NUDT2:-1.36<br>NUDT3: -1.65<br>NUDT4: +1.37<br>NUDT10:-1.29<br>NUDT13: +1.21<br>NUDT22: -1.36	NUDT4: +1.66<br>NUDT5: +1.68<br>NUDT9: +1.66<br>NUDT11: +1.70<br>NUDT17: +1.52<br>NUDT17: -1.54<br>NUDT19: +1.80<br>NUDT6: +2.55
NES			
COG8	COG1: +1.21<br>COG2: +1.23<br>COG5: +1.26<br>COG5: -1.33<br>COG6: -1.45		COG1: -1.70<br>COG2: -2.85
S100A9			S100A12: +1.61<br>S100A16: +1.20
SCG2	SCG3: +1.31	SCG2: +1.23<br>SCG3: -1.31	SCG5: +1.85
NRP1	NRP1: -1.40<br>NRP2: -1.63	NRP1: -1.37<br>NRP2: +1.23	NRP2: +1.234<br>NRP2: -2.011
IGLC2	IGL7: +1.37<br>IGLL5: +1.22<br>IGLC7: +1.36		IGLL1: -1.77<br>IGLV140/44: -1.84

	IGLV@: +1.24<br>IGLV1-40: +1.62		
PCDHGC5	PCDHGC5: -1.50<br>PCDGC3: +1.44<br>PCDHA10: +1.20<br>PCDHGA8: -1.35<br>PCDH12: -1.76<br>PCDHGB7: -2.21	PCDHB5: +1.20<br>PCDH11X: -1.26<br>PCDHB10: -1.34<br>PCDH12: -1.38	PDCDH17: +3.75<br>PDCDH18: +1.68<br>PDCDHGA9: +1.64<br>PDCDH19: +1.51<br>PCDH7: -1.53 //-1.70
FABP3	FABP5. +1.34		FABP7: -1.70
SERPINA1	SERPINA1: -2.02	SERPINA1: -1.28	SERPINA6: +2.90
HSPB1		HSPA8:+1.34<br>HSPA9:+1.19<br>HSPB9:+1.21<br>HSPD1:+1.26<br>HSP90A1:1.26	HSPB7: +1.54<br>HSPB8: -2.64
GPT			GPT:+2.03
CLIC6			CLIC2: -1.96
CACNG8	CACNG8: +1.22	CACNG8: +1.23<br>CACNG2: +1.24	CACNG1: +1.89<br>CACNGG3: -1.51
SMARCE1	SMARCA4: +1.20<br>SMARCA1: +1.18		SMARCA2: +1.80<br>SMARCA2: +1.35
KNG1	KNG1: +1.26		KNG1: +1.82
APOA2	APOB: 1.34<br>APOC2: -1.65<br>APOBEC3A: -1.46 (cytidine deaminase editing enzyme, antiviral)<br>APOOL: -1.70	APOE: +1.19<br>APOOC: +1.19<br>APOC2: -1.30<br>APOBEC2: -1.60 (Editing enzyme of ApoE mRNA)	APOA2: +1.86
WIPF3	WIPF1: +1.46		WIPF2: +1.21
RAB3D	RAB3IP: -1.39<br>RAB33A: -1.45	RAB3B:+1.19<br>RAB36:+1.20<br>RAB39A: +1.29<br>RAB3IL1:+1.26<br>RAB3GAP1: -1.30	RAB3C: -1.70<br>RAB30: +3.01<br>RAB37: +2.06
ALBUMIN			ALBUMIN: +2.55
PVALB			
AHS6	AHS2: +1.19		
CRMP1			
VITAMIN D BINDING PROTEIN	VDR: +1.19<br>VDR: +1.18		
IGHG1	IGHG1: -1.35//-2.68//+1.66		IGHG1: 1.72//1.51//1.45
PVRL1		PVRL1:-1.26	PVRL1: -3.45
SHISA6	SHISA3: +1.80<br>SHISA5: +1.21	SHISA3: +1.22<br>SHISA5: +1.32	SHISA2: -1.51
